# Exploring Similarities and Differences Between Methods That Exploit Patterns of Local Genetic Correlation to Identify Shared Causal Loci Through Application to Genome‐Wide Association Studies of Multiple Long Term Conditions

**DOI:** 10.1002/gepi.70012

**Published:** 2025-06-19

**Authors:** Rebecca Darlay, Rupal L. Shah, Richard M. Dodds, Anand T. N. Nair, Ewan R. Pearson, Miles D. Witham, Heather J. Cordell, ADMISSION Research Collaborative, Victoria Bartle, Heather J. Cordell, Ray Holding, Tom Marshall, Fiona E. Matthews, Paolo Missier, Ewan R. Pearson, Elizabeth Sapey, Thomas Scharf, Mervyn Singer, James M. S. Wason, Rachel Cooper, Chris Plummer, Sian M. Robinson, Avan A. Sayer, Miles D. Witham

**Affiliations:** ^1^ Population Health Sciences Institute, Faculty of Medical Sciences Newcastle University Newcastle Upon Tyne UK; ^2^ Translational and Clinical Research Institute, Faculty of Medical Sciences Newcastle University Newcastle upon Tyne UK; ^3^ Division of Diabetes Endocrinology and Reproductive Biology University of Dundee Dundee UK; ^4^ NIHR Newcastle Biomedical Research Centre, Newcastle upon Tyne Hospitals NHS Foundation Trust, Tyne and Wear NHS Foundation Tust and Newcastle University, Newcastle upon Tyne, UK; ^5^ Public Coinvestigator, ADMISSION Research Collaborative Newcastle upon Tyne UK; ^6^ AGE Research Group, Translational and Clinical Research Institute, Faculty of Medical Sciences Newcastle University Newcastle upon Tyne UK; ^7^ Institute of Applied Health Research University of Birmingham Birmingham UK; ^8^ Research and Enterprise Office University of Hull Hull UK; ^9^ School of Computer Science University of Birmingham Birmingham UK; ^10^ Division of Population Health and Genomics, Ninewells Hospital and School of Medicine University of Dundee Dundee UK; ^11^ Digital Services, Newcastle upon Tyne Hospitals NHS Foundation Trust Newcastle upon Tyne UK; ^12^ PIONEER Hub University of Birmingham Birmingham UK; ^13^ Institute of Inflammation and Ageing University of Birmingham Birmingham UK; ^14^ University College London Hospitals NHS Foundation Trust London UK; ^15^ Bloomsbury Institute for Intensive Care Medicine University College London London UK; ^16^ Biostatistics Research Group, Population Health Sciences Institute Newcastle University Newcastle upon Tyne UK

## Abstract

Genetic correlation analysis can provide useful insight into the shared genetic basis between traits or conditions of interest. However, most genome‐wide analyses only inform about the degree of global (overall) genetic similarity and do not identify the specific genomic regions that give rise to this similarity. Identification of the key genomic regions contributing to shared genetic correlation between traits could allow the genes in these regions to be prioritised for investigation of potential shared biological mechanisms. In recent years, several statistical tools (e.g. LAVA, ρ‐HESS, SUPERGNOVA and LOGODetect) have been developed to investigate local (in contrast to global) genetic correlation. These tools partition the genome into multiple segments and provide estimates of the genetic correlation captured by each individual segment. We applied these tools to publicly available European ancestry genome‐wide association study (GWAS) summary statistics for three pairs of commonly occurring conditions: hypertension with atrial fibrillation and flutter, hypertension with chronic kidney disease, and hypertension with type 2 diabetes. Despite each of the methods aiming to address the same question, the results were found to be inconsistent across tools, with some identified regions overlapping and others implicated only by a single tool. Computer simulations using genetic data from UK Biobank, carried out under known generating conditions, suggest that LAVA and, to a lesser extent, ρ‐HESS, provide the most reliable identification of genuine shared genetic factors. A newly‐developed tool, HDL‐L, also performed highly competitively. Here we highlight the similarities and differences between the results obtained from these methods and discuss some potential reasons underlying these differences.

## 1 Introduction

Multiple long‐term conditions (MLTC; commonly referred to as multimorbidity) currently represent one of the most pressing challenges in medical research, practice and public health (Whitty et al. 2020). MLTC, usually defined as the coexistence of two or more long‐term health conditions (Kingston et al. 2018; Violan et al. 2014), are common and has been estimated to affect around 37% of the global adult population (Chowdhury et al. 2023), with the prevalence projected to rise over the next 20 years, particularly among older people (Kingston et al. 2018).

MLTC are particularly common among people admitted to hospital and are thus of critical importance to hospital‐based healthcare systems, however comparatively little research has focused on the underlying causes, consequences, and care of MLTC in hospital settings. To help address this gap in knowledge, the ADMISSION Research Collaborative, based across five UK academic centres, was created in 2021. The ambition and scope of ADMISSION is wide (Cooper et al. 2024; Witham et al. 2025), including both qualitative and quantitative research focused specifically on patients with MLTC who are admitted to the hospital. A key area of interest within ADMISSION is the extent to which there are common biological mechanisms underpinning shared conditions, and the extent to which this fact might be useful in informing treatment and management of those conditions (Langenberg et al. 2023).

As a first step towards this goal, we decided to investigate the extent to which genetic correlation analysis might provide useful insight into the shared genetic basis between traits or conditions of interest. Estimation of genome‐wide correlation between traits, using software tools such as GCTA (Yang et al. 2011) or LDSC (B. K. Bulik‐Sullivan et al. 2015), is now ubiquitous (B. Bulik‐Sullivan et al. 2015; Wu et al. 2022) and can imply the existence of shared genetic and biological mechanisms. However, pinpointing the specific underlying shared genetic factors can be challenging. In recent years, several statistical methods and accompanying software tools, including ρ‐HESS (Shi et al. 2017), SUPERGNOVA (Zhang et al. 2021), LOGODetect (Guo et al. 2021) and LAVA (Werme et al. 2022), have been developed to investigate *local* (in contrast to *global*) genetic correlations. These tools partition the genome into multiple segments and provide estimates of the genetic correlation captured by each individual segment, thus allowing a more nuanced view regarding which portions of the human genome (and thus which potential genes) contribute to the overall genetic correlation.

We applied these tools to pairs of conditions that had previously been identified to cluster together within hospitalised patients (Robertson et al. 2022). We focussed on three pairs of conditions from Table 4 of Robertson et al. (2022) that were considered of particular relevance within the context of the wider ADMISSION project, and for which summary statistics from genome‐wide association studies (GWAS) were readily available, namely: hypertension together with atrial fibrillation and flutter; hypertension together with chronic kidney disease; and hypertension together with diabetes. We also applied these same tools to simulated traits, simulated under known generating models in terms of their shared genetic causes, in an effort to understand the differences between the tools and the results they generated, and to determine which set(s) of results might be considered the most reliable.

## 2 Methods

### 2.1 Data Sets

Genome‐wide association study (GWAS) summary statistics were downloaded from the relevant repositories for the four study phenotypes of interest: hypertension (http://geneatlas.roslin.ed.ac.uk/downloads/?traits=266) (Canela‐Xandri et al. 2018), atrial fibrillation and flutter (https://csg.sph.umich.edu/willer/public/afib2018/) (Nielsen et al. 2018), chronic kidney disease (CKD) (https://ckdgen.imbi.uni-freiburg.de/datasets/Wuttke_2019) (Wuttke et al. 2019) and type 2 diabetes (https://diagram-consortium.org/downloads.html) (Mahajan et al. 2022). All data sets were filtered for minor allele frequencies > 0.05 and, where relevant, INFO scores > 0.9. For the hypertension cohort, there were 84,910 cases, as specified by UK Biobank classification field I10‐I15 Hypertensive diseases (Bycroft et al. 2018), and 367,354 population controls, typed at 8,081,164 variants. In the atrial fibrillation data set, there were 60,620 cases with atrial fibrillation and flutter and 970,216 controls typed at 11,427,610 variants. For the CKD cohort, where cases were defined as having an estimated glomerular filtration rate (eGFR) below 60 mL min^−1^ per 1.73 m^3^, there were 41,395 cases and 439,303 controls with 8,930,073 variants. Finally, the diabetes cohort was restricted to 80,154 cases specifically with type 2 diabetes, and 853,816 controls, with 8,735,477 variants. Table S1 summarises these data sets.

### 2.2 Genetic Correlation Analysis

Four local genetic correlation analysis programs—ρ‐HESS (Shi et al. 2017), SUPERGNOVA (Zhang et al. 2021), LOGODetect (Guo et al. 2021) and LAVA (Werme et al. 2022)—were applied to the three pairs of conditions of interest, namely hypertension paired with each of the other three traits (atrial fibrillation and flutter, CKD, type 2 diabetes), in turn. Results from the four analysis packages were compared and regions of interest were examined by plotting in LocusZoom (Pruim et al. 2010). Below we give a brief summary of the methodology implemented in each of the packages; for full details please see the relevant original publications.

#### 2.2.1 ρ‐HESS

This method uses GWAS summary data to identify genome segments harbouring local genetic correlation—that is, correlation between traits due to typed genetic variants from within the small segment—while allowing for overlapping GWAS samples between the traits and linkage disequilibrium (LD) among variants. Segments are defined by partitioning the genome into 1703 approximately independent LD regions each of width ~1.6 Mb. (Our analyses used the 1690 of these regions for which summary statistics were available in our data sets). Using techniques similar to those used in cross‐trait LD score regression (LDSC) (B. Bulik‐Sullivan et al. 2015), ρ‐HESS first obtains estimates of the local SNP‐heritability of each trait within each segment before calculating local genetic correlation estimates for those segments with significant local SNP‐heritability. Truncated singular value decomposition (SVD) is used to remove noise in the externally‐estimated LD matrix and reduce the standard error of the estimates of local genetic correlation. In our application, each trait pair was analysed separately with the threshold for statistical significance set at *p* < 0.05/1690 = 2.96E‐5, based on a Bonferroni correction for the 1690 ρ‐HESS‐defined test loci.

#### 2.2.2 SUPERGNOVA

Like ρ‐HESS, SUPERGNOVA uses GWAS summary data to identify genome segments harbouring local genetic correlation between two complex traits, while allowing for overlapping GWAS samples and LD among variants. SUPERGNOVA first decorrelates the local summary statistics (z scores) with eigenvectors of the local LD matrix, as estimated from the 1000 Genomes Project (Auton et al. 2015) external reference panel. After decorrelation, the local genetic covariance is estimated through a weighted least squares regression in each region. Previous work (Zhang et al. 2021) suggested that SUPERGNOVA outperformed ρ‐HESS in application to simulated data, showing higher power and lower bias, although the reasons for its superior performance were not clearly delineated. In our application, each pair of traits was tested with the significance threshold set at *p* < 0.05/2353 = 2.12E‐05, based on a Bonferroni correction for the 2353 SUPERGNOVA‐defined test loci.

#### 2.2.3 LOGODetect

This method uses so‐called scan statistics to identify genome segments harbouring local genetic correlation between two complex traits. Compared to other methods, LOGODetect does not prespecify candidate regions of interest, and instead scans local regions of varying sizes, automatically detecting those regions with evidence of shared genetic components. The scan statistic for region R, Q(R), is a LD score‐weighted inner product of local z‐scores from two GWAS and is conceptually similar to local genetic correlation. Regions with high absolute values of Q(R) show concordant association patterns across multiple SNPs in the region and the sign of Q(R) indicates if the correlation is positive or negative. We applied LOGODetect to each of our trait pairs sequentially. LOGODetect first examines 204 LOGODetect‐defined LD blocks separately and identifies local regions with *p* < 0.05. These regions are then aggregated across the LD blocks and adjusted for multiple testing, and only statistics for those local regions passing the in‐software significance threshold are reported. We set the LOGODetect *p*‐value cut‐off as unadjusted *p* < 0.05/204 = 2.45E‐04.

#### 2.2.4 LAVA

LAVA is an R package that, in addition to estimating the standard bivariate local genetic correlation between two phenotypes (binary or continuous)—while accounting for known or estimated sample overlap—can also test the local heritability for all phenotypes of interest and analyse conditional genetic relationships between traits using partial correlation or multiple regression. A sample overlap file is optional, which can be calculated using the results from cross‐trait analysis with LDSC. A comparison of the theoretical differences between LAVA, ρ‐HESS and SUPERGNOVA (Werme et al. 2022) suggested that, in additional to small differences related to construction of the estimators, some key differences are (a) unlike LAVA, both ρ‐HESS and SUPERGNOVA make no specific accommodations for binary phenotypes; (b) ρ‐HESS and LAVA use different numbers of principal components when inverting the externally‐estimated LD matrix; (c) ρ‐HESS constructs its test statistics assuming that the sampling distributions of the local genetic correlation and covariance estimates are normal, while LAVA uses a simulation‐based approach to directly generate p‐values; and (d) SUPERGNOVA takes a random‐effects perspective (in contrast to the fixed‐effects perspective of ρ‐HESS and LAVA) and thus ends up estimating a fundamentally different quantity, with a different interpretation, from that estimated by ρ‐HESS and LAVA.

To obtain a local correlation score between all trait pairs for each of the 2495 test loci within the LAVA‐supplied loci (regions) file, the univariate p value threshold was set to 1, so that no regions were filtered out by LAVA. Sample overlap between all possible trait pairs was calculated and included in the model. The threshold for statistical significance of the resulting local genetic correlation estimates between each trait pair was set to *p* < 0.05/2495 = 2E‐5.

#### 2.2.5 HDL‐L

While this manuscript was in revision, a new method (and R package) for local genetic correlation analysis, high‐definition likelihood (local version) (HDL‐L), was published, which the authors found to outperform LAVA in terms of estimation efficiency, accuracy and reliability (Li et al. 2025). We therefore repeated our analyses of the real and simulated traits using HDL‐L, and compared the results with those previously obtained using LAVA, ρ‐HESS, SUPERGNOVA and LOGODetect.

### 2.3 Simulation Study

GCTA (Yang et al. 2011) was used to simulate three sets of case‐control phenotypes, based on imputed genotype data from UK Biobank (Bycroft et al. 2018) participants. After excluding individuals with non‐European ancestry, gender mismatches and those with close relatives within the cohort, 276,257 individuals were randomly assigned to three nonoverlapping groups each of size 92,000. The genotype data for these individuals were extracted to generate three trait‐specific genotypic data sets (A, B, C) each containing 5,554,261 autosomal, well imputed SNPs (INFO score > 0.3 and MAF > 0.01). From these, nine causal SNPs were assigned to operate in each trait, as listed in Table 1. For each trait (A, B, C), three separate unique trait‐causing SNPs were chosen, all situated in unique genomic regions. There were also three regions (on chromosomes 2, 10, and 12) where the same variant was chosen to operate on two traits, and one region (on chromosome 1) where the same casual variant operated on all three traits. In addition, there were three regions (on chromosomes 2, 19, and 22) where a pair of traits had causal SNPs that were close to, but not in LD with, each other. Finally one region (on chromosome 4) was chosen where all three traits had causal SNPs that were close to, but not in LD with, one another. Three nonoverlapping sets of 9000 cases and 36,000 controls were simulated from these three trait‐specific genotypic data sets. GWAS summary statistics were then generated for each trait by using logistic regression with PLINK 1.9 (Chang et al. 2015).

**Table 1:** Assigned causal SNPs for each simulated trait (A, B, and C), with resulting odds ratio (OR) and *p* value from logistic regression analysis carried out in PLINK.

Causal SNPs	Trait A					Trait B					Trait C				
Scenario	Chr	SNP	BP	OR	*p*	Chr	SNP	BP	OR	*p*	Chr		BP	OR	*p*
Unique	8	rs10956403	129007207	0.832	1.06E‐24	5	rs7726834	52512986	1.699	1.35E‐203	3	rs56760958	85983138	1.422	1.26E‐96
	14	rs10148839	98779059	1.193	2.79E‐23	7	rs215600	32333642	0.832	6.74E‐25	10	rs12356635	5026504	0.7738	6.66E‐35
	16	rs72814388	87906000	1.125	1.97E‐09	9	rs10821469	93257245	0.668	8.50E‐94	18	rs12960274	51285499	0.889	4.41E‐10
Shared	1	rs6703265	76076390	1.481	3.09E‐112	1	rs6703265	76076390	0.866	2.74E‐15	1	rs6703265	76076390	1.464	7.97E‐107
	2	rs7587559	135069472	0.569	9.20E‐156	—	—	—	—	—	2	rs7587559	135069472	1.387	6.08E‐72
	10	rs4750613	15293155	1.523	2.81E‐113	10	rs4750613	15293155	1.436	3.14E‐82	—	—	—	—	—
	—	—	—	—	—	12	rs10849657	119845498	0.568	1.64E‐124	12	rs10849657	119845498	0.819	2.62E‐20
Shared	4	rs3775226	87989465	0.633	2.69E‐85	4	rs13118664	88239609	1.224	1.48E‐27	4	rs4693818	88421277	1.33	1.52E‐45
location	—	—	—	—	—	2	rs1659676	27399294	0.828	8.30E‐24	2	rs1260326	27730940	1.652	4.18E‐192
	19	rs2304128	19746151	1.612	2.25E‐66	—	—	—	—	—	19	rs10412710	19993507	1.183	3.00E‐19
	22	rs738409	44324727	1.214	2.08E‐23	22	rs5764296	44181312	1.141	3.19E‐14	—	—	—	—	—

### 2.4 Colocalisation Analysis

GWAS summary statistics, converted to Approximate Bayes Factors (Wakefield 2009), can be used within the coloc package (Giambartolomei et al. 2014) to perform genetic colocalisation analysis of two potentially related phenotypes, to ask whether they share common genetic causal variant(s) in a given region. The posterior probabilities of association of any given SNP with each trait are calculated, as are the posterior probabilities of both traits sharing an association. From these, support can be estimated for the following hypotheses: H_0_: neither trait has a genetic association in the region; H_1_: only trait 1 has a genetic association in the region; H_2_: only trait 2 has a genetic association in the region; H_3_: both traits are associated, but with different causal variants; or H_4_: both traits are associated and share a single causal variant. The most recent version of coloc (Wallace 2021) uses the SuSiE (Wang et al. 2020) framework for fine mapping in the presence of multiple causal variants, allowing the single variant assumption of coloc to be relaxed if an LD matrix is provided.

Regions that had been identified as significantly correlated between real data sets by LAVA were taken forward for analysis with coloc (including the SuSiE functionality) to tease out the nature of the correlation and identify the SNPs driving the colocalisation signal.

## 3 Results

### 3.1 Initial Findings With All Four Tools

**Figure 1:**
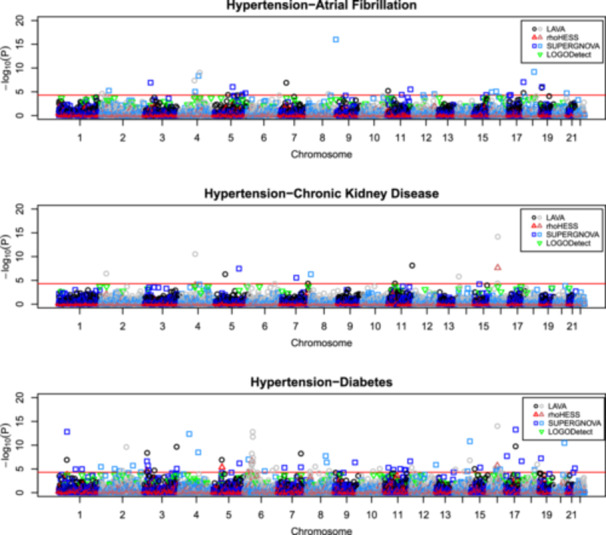
A comparison of regions of local correlation as detected by LAVA, rho‐HESS, SUPERGNOVA and LOGODetect in the three ADMISSION trait‐pairs. The red line indicates a nominal significance threshold of *p* < 5E‐5.

Despite each of the four local correlation packages aiming to address the same question, there was little consistency seen across tools (Figure 1 and Table 2), with some identified regions overlapping across tools and others implicated by only a single tool.

**Table 2:** All statistically significant regions (LAVA *p* < 2E‐05; ρ‐HESS *p* < 2.96E‐05; SUPERGNOVA *p* < 2.12E‐05; LOGODetect *p* < 2.45E‐04) of local genetic correlation as determined by each tool, in each ADMISSION trait‐pair.

Trait pair	Region	Chr	Start	Stop	LAVA *p*	rhoHESS *p*	SUPERGNOVA *p*	LOGODetect *p*
HT‐AF	1	1	10768002	10808823				1.99E‐04
	2	1	218471383	218546170				1.99E‐04
	3	2	37008066	38882278			5.89E‐06	
	4	2	65234613	65395946				1.99E‐04
	5	3	26393305	27606695			1.23E‐07	
	6	4	79880102 81154587	81206182 81207039	4.28E‐08			1.99E‐04
	7	4	83017789	83994716			9.84E‐06	
	8	4	103388441	104802530			4.40E‐09	
	9	4	109978983 111552398	111733579 11719685	9.41E‐10			1.99E‐04
	10	4	174439702	174471560				1.99E‐04
	11	5	32672832	32837647				1.99E‐04
	12	5	105887333	106800282			9.56E‐07	
	13	5	172377031	173611384			1.82E‐05	
	14	7	25671576 27217359	27351286 27321357	1.26E‐07			1.99E‐04
	15	8	141358681	143750205			1.03E‐16	
	16	11	1857846 1880811	2477449 2055198	6.29E‐06			1.99E‐04
	17	11	16234918	16352545				1.99E‐04
	18	11	120173354	121660636			3.09E‐06	
	19	13	22284926	22320658				1.99E‐04
	20	15	80991447	81080488				1.99E‐04
	21	16	598838	945806			1.20E‐05	
	22	16	15885866	16220858			8.51E‐06	
	23	16	53794830	53850741				1.99E‐04
	24	16	73002421	73066954				1.99E‐04
	25	17	76596288	77412786	1.72E‐05			
			76446689	77093768			9.32E‐08	
	26	18	46515916	46528065				1.99E‐04
	27	18	47816777 48480841	48784027 48796006			6.54E‐10	1.99E‐04
	28	19	3085447	3893909	1.42E‐06			
	29	19	4349416	5191547			9.30E‐07	
	30	20	57320545	58295563			2.00E‐05	
HT‐CKD	31	1	243336384	243505047				1.99E‐04
	32	2	26894103 26897501	28819510 26924823	3.67E‐07			1.99E‐04
	33	4	77182726	77421357				1.99E‐04
	34	4	79880102 81154232	81206182 81205868	2.91E‐11			1.99E‐04
	35	4	156412234	156743305				1.99E‐04
	36	5	55221399	55968966	5.04E‐07			
	37	5	124403478	125266684			3.39E‐08	
	38	5	176740704	176836532				1.99E‐04
	39	6	43801878	43823694				1.99E‐04
	40	7	77102731	77953248			2.73E‐06	
	41	7	151390387	151423345				1.99E‐04
	42	8	1625732	2042364			5.18E‐07	
	43	11	30749936	30788677				1.99E‐04
	44	11	124403478	125266684	7.33E‐09			
	45	14	22760701	23985936	1.55E‐06			
	46	16	20351937 20351262	20418137 22667048	6.56E‐15			1.99E‐04
			20150571	22448904		2.35E‐08		
	47	17	59421778	59492920				1.99E‐04
HT‐T2D	48	1	38474037 39537291 39549152	40200950 40933221 40076517	1.21E‐07		1.59E‐13	1.99E‐04
	49	1	87654606	89447978			1.14E‐05	
	50	1	120347632 120434793	144993501 120528568			1.11E‐05	1.99E‐04
	51	1	201755933	201885285				1.99E‐04
	52	2	11320 62115	1158981 659384			3.65E‐06	1.99E‐04
	53	2	65228236 65246123	66374803 65403970			9.47E‐06	1.99E‐04
	54	2	121302804	121341281	2.21E‐05^a^			1.99E‐04
	55	2	144503689	145977920	2.42E‐10			
	56	2	180662102	181627195			2.03E‐06	
	57	2	227066471	227205605				1.99E‐04
	58	3	11997659	12859209	4.18E‐09			
			11019665	13070799		2.14E‐05		
			11221721	12858028			2.36E‐07	
	59	3	14816745	16661587			1.96E‐06	
	60	3	184524269 185263467 185445144	185709996 186780913 185550881	2.21E‐10		8.84E‐06	1.99E‐04
	61	4	38380354	38423520				1.99E‐04
	62	4	48227642	53412129			4.26E‐13	
	63	4	103388441	104802530			3.33E‐09	
	64	5	55221399 55417349 55794559	55968966 56621102 55902375	1.21E‐07	4.68E‐06		1.99E‐04
	65	5	157199547	158491655			6.87E‐07	
	66	6	20231516 20470908	22049758 20774596	2.61E‐05^a^		1.01E‐07	1.99E‐04
	67	6	31250557	31320268	8.65E‐07			
	68	6	31320269	31427209	1.69E‐06			
	69	6	31427210	32208901	6.07E‐09			
			31571218	32682664		2.36E‐07		
	70	6	32208902	32454577	7.53E‐07			
	71	6	32454578	32539567	1.51E‐13			
	72	6	32539568	32586784	2.70E‐07			
	73	6	32586785	32629239	1.75E‐12			
	74	6	32682214	32897998	4.47E‐08			
	75	6	43756863	4380488				1.99E‐04
	76	6	163336362	164687462	5.68E‐06			
	77	7	28039590 28131885	28710596 28224053			5.50E‐06	1.99E‐04
	78	7	130418705	131856481	5.85E‐09			
			130075370 130423490	130797123 130467446			4.30E‐06	1.99E‐04
	79	8	29744541	31134786	3.34E‐06			
	80	8	95810772	96533604			1.87E‐08	
	81	8	101451169	102937063			4.07E‐07	
	82	8	118185938	118240824	2.52E‐05^a^			1.99E‐04
	83	9	21999800	22134172				1.99E‐04
	84	9	125545194	126926376			4.46E‐07	
	85	10	80922957	81021543				1.99E‐04
	86	10	114255955 114743019	115588903 114823426	9.28E‐06			1.99E‐04
	87	11	14936943	16789155			5.41E‐06	
	88	11	42854878 43629150	43952796 43882764	7.75E‐05^a^		1.43E‐05	1.99E‐04
	89	11	82847086	83575703			9.82E‐06	
	90	11	106252171	107489662			1.55E‐05	
	91	11	113105405	113958177			1.88E‐05	
	92	11	132386834	133679495			2.39E‐07	
	93	12	3069507	3871114	5.48E‐06			
	94	12	54392449	544446495				1.99E‐04
	95	12	132807034	133841558			1.33E‐06	
	96	14	23985937	24906056	1.27E‐05			
	97	14	102004378	103617472	1.58E‐07			
			102341650	104759919			1.54E‐11	
	98	15	37962916	39238840	1.01E‐05			
	99	15	53077634	53165635				1.99E‐04
	100	15	62737452	64335240			5.11E‐06	
	101	16	20344077	20392391				1.99E‐04
	102	16	27443062	29043177	6.66E‐06			
	103	16	53393883	54866095	9.52E‐15			
			53382572	55903774		2.15E‐06		
			51703888 53795636	53845487 53850868			1.38E‐05	1.99E‐04
	104	16	68429894	70885818			1.27E‐05	
	105	17	3426133	4321755			1.98E‐08	
	106	17	45883902	47516224	1.75E‐10			
			46828412 46957987	48027295 47146515			5.18E‐14	1.99E‐04
	107	17	76446689	77093768			2.34E‐07	
	108	18	57505073 57726627	58816387 58055105	2.76E‐05^a^		1.57E‐05	1.99E‐04
	109	18	69311743	70540349	1.15E‐06			
	110	19	4349416	5191547			5.84E‐08	
	111	20	50635347	51389135			3.27E‐11	
	112	21	44690856	45598522			7.54E‐06	

Abbreviations: AF, atrial fibrillation and flutter; CKD, chronic kidney disease; HT, hypertension; T2D, type 2 diabetes.

^a^ Loci reported with close‐to threshold *p* values in LAVA where significant with other tools.

For the first pair of traits (hypertension paired with atrial fibrillation and flutter), while LAVA identified six regions of local genetic correlation, ρ‐HESS found no local correlations, and, of the 14 SUPERGNOVA‐identified regions, only one at chr17:76446689‐77093768 overlapped with other tools (*p* = 1.72E‐05 for LAVA; *p* = 9.32E‐08 for SUPERGNOVA). There were 16 regions where *p* < 2.45E‐4 was reported by LOGODetect, some of which overlapped with LAVA findings and others of which were distinct.

There were a total of 17 potential regions of local genetic correlation detected between hypertension and CKD. LAVA found six of these, of which one on chr16:20351262‐22667048 (*p* = 6.56E‐15) was also supported by rho‐HESS. No other ρ‐HESS results were significant, and SUPERGNOVA reported an additional three unrelated regions, not equivalent to those detected by LAVA. There were 11 regions where *p* < 2.45E‐4 was reported by LOGODetect, some of which overlapped with LAVA findings and others of which were distinct.

For the hypertension‐diabetes pairing, there were considerably more significant results reported by LAVA and SUPERGNOVA than for the other two trait‐pair analyses. LAVA identified a total of 25 significant regions, of which 8 occurred within the Major Histocompatibility Cluster (MHC) on chromosome 6. It is possible that these MHC signals indicate contamination in the labelling of disease in type 2 diabetes data sets (and thus GWAS summary statistics) with patients that actually have type 1 diabetes. Alternatively, it might be that other autoimmune conditions are driving both hypertension and diabetes (e.g., via chronic inflammation, or obesity, or some other intermediate phenotype).

ρ‐HESS identified two of the hypertension‐diabetes LAVA signals, on chromosomes 5 and 6, plus a region on chromosome 3 supported by SUPERGNOVA and one on chromosome 16 supported by SUPERGNOVA and LOGODetect. SUPERGNOVA reported 33 regions in total, 10 of which overlapped with LAVA significant or close‐to‐significant regions. LOGODetect found 25 regions of local correlation below the *p* < 2.45E‐4 significance threshold, some of which overlapped with LAVA findings and others of which were distinct.

To investigate whether the inconsistencies seen between the results from LAVA, ρ‐HESS, SUPERGNOVA and LOGODetect might be attributable to the different partitions of the genome into test regions (or test segments/loci) used by the programs, we attempted to force the programs to use the exact same partitions. This was only possible for LAVA, ρ‐HESS and SUPERGNOVA, for which it is possible to replace the program's default partitions with alternative user‐specified partitions. We used the three default partitions from LAVA, ρ‐HESS and SUPERGNOVA and applied them to the hypertension‐diabetes data using each of the three packages in turn. Figure S1 shows that, regardless of the partition definition used, the results from LAVA (top panel) are largely consistent, albeit with different levels of significance achieved depending on the partition definition used. Similarly, for ρ‐HESS (middle panel) and SUPERGNOVA (bottom panel), the partition definition affects the overall level of significance achieved but not the location of the regions of significant genetic correlation identified. Figure S2 displays the same results in a different way, focussing on comparing the results from the three different packages when using either the LAVA default partitions (top panel), the ρ‐HESS default partitions (middle panel) or the SUPERGNOVA default partitions (bottom panel). Similar to the results seen for the hypertension‐diabetes pairing when using the default partitions (Figure 1 lower panel), we find that, although there are a few loci where consistency can be seen among the tools, by and large the results are not particularly concordant, with many regions implicated only by a single tool. Thus, the differences seen in the original analyses (which used the default genomic partitions for each program) can, at most, be only partially attributed to the differences in the partitioning.

Although the differences in partitions used did not generally affect the presence or absence of a signal, we do see quite substantial differences in the magnitude of the p‐values between the different partitions. To explore this in more depth, Table S2 shows the results from LAVA, using the default partitions from either LAVA, ρ‐HESS or SUPERGNOVA, from a selection of colocalising regions in the hypertension and type 2 diabetes data sets. This gives numbers of SNPs and window position and size for each partition at each locus, as well as the reported local genetic correlation (rho) with upper and lower confidence limits and the associated *p*‐value. Differences in the p‐values appear to be primarily driven by differences in the partitions and thus the SNPs (and number of SNPs) that are captured.

This is further explored in Figure S3, which shows the underlying GWAS associations for hypertension and diabetes in two different regions, the first on chromosome 1 and the second on chromosome 5. The limits of the default windows are shown for LAVA (red), ρ‐HESS (blue), and SUPERGNOVA (green). For the chromosome 1 region, the windows in each partition set cover the full genome‐wide significant locus for both traits, thus capturing all relevant information, resulting in quite similar significance in terms of the LAVA *p*‐values obtained using the diffferent window definitions. For chromosome 5, LAVA gives substantially different results across the different partition windows, which is probably due to the fact that the different partitions capture different portions of the significant locus. The LAVA and SUPERGNOVA partitions both miss an additional peak of significance seen to the right of the main locus, whereas the ρ‐HESS partition has a wider window and captures the full region.

### 3.2 Simulation Results

Given the relatively inconsistent results between tools seen in the real data sets for each trait pairing, we undertook a simulation study as described in the Methods to assess how well each tool worked in a number of different scenarios.

#### 3.2.1 Same Causal SNP

**Figure 2:**
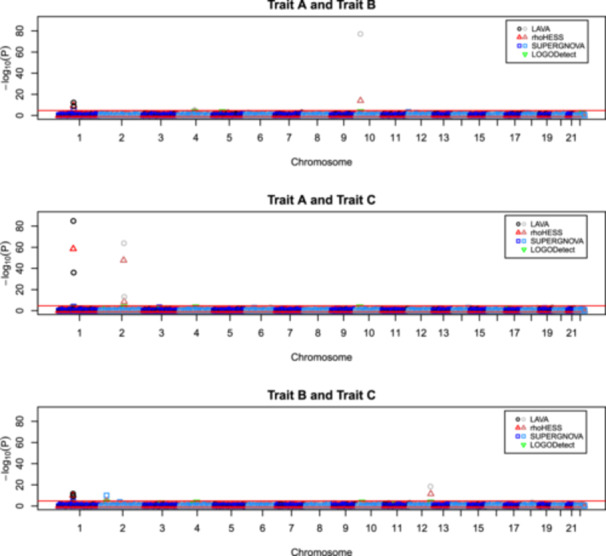
A comparison of regions of local correlation as detected by LAVA, rho‐HESS, SUPERGNOVA and LOGODetect in the three simulated trait‐pairs. The red line indicates a nominal significance threshold of *p* < 5E‐5.

Based on the simulation models (Table 1), Traits A and B shared causal SNPs on chromosomes 1 and 10, traits A and C shared causal SNPs on chromosomes 1, and 2, and traits B and C shared causal SNPs on chromosomes 1 and 12. Both LAVA and ρ‐HESS successfully detected statistically significant regions of local correlation between all expected pairs (Table S3 and Figure 2), with LAVA achieving the strongest levels of significance (smallest *p* values). LOGODetect also reported these same regions, but the local correlation at 1:76076390:G:A was only detected between traits A and C. SUPERGNOVA performed arguably the least well, detecting the local correlation between traits B and C at 12:119845498:T:A, but not that between traits A and B at 10:15293155:C:T or between traits A and C at 2:135069472:G:A. SUPERGNOVA also only reported a borderline‐significant local correlation at 1:76076390:G:A for all three trait pairs, in contrast to the compelling significance levels achieved by LAVA and ρ‐HESS.

All software packages agreed on the sign of the genetic correlation (*r*_g_), but the magnitude of the *r*_g_ reported by LAVA was considerably greater than that reported by ρ‐HESS and SUPERGNOVA. We hypothesise that differences in the magnitude of *r*_g_ and/or the significance levels obtained, resulting in more or less concordance seen between different methods for different trait pairs at different loci, most likely result from a complex interplay between the trait‐specific patterns of association (which are heavily influenced by the LD pattern in the region), the extent to which these are captured by the window used by a specific method, and underlying methodological differences between the packages. No unexpected regions of local correlation were detected by any of the packages.

#### 3.2.2 Different Causal SNP, Same Region

Four regions (on chromosomes 2, 4, 19, and 22) were chosen so that each trait pair shares a causal SNP within the same 1 Mb region, but not the same SNP. These different SNPs were also selected to not be in strong LD with each other. The performance of the four tools was somewhat variable under these conditions (Table S4 and Figure 2). LOGODetect reported significant local correlation for 5 out of 6 possible trait pairings, whereas ρ‐HESS reported borderline significant local correlation for two pairings, however not all of these results passed the strict significance threshold. Both LAVA and SUPERGNOVA identified a region of local correlation at 2:27399294 between traits B and C. Figure S4 shows that the causal SNPs chosen for this locus are, in fact, in weak LD in the 1000 Genomes European reference population (*r* ^2^ < 0.4 as shown by LocusZoom), indicating that these tools may be identifying regions of local correlation due to LD rather than an underlying shared genetic cause. LAVA also indicated the region around 4:87989465 to be locally correlated between traits A and B, but not between other trait pairings. The estimated local genetic correlations and p‐values achieved by LAVA were considerably less than had been achieved for the loci where the same causal SNP had been operating. The direction of correlation was consistent between tools where a region had been identified as statistically significant, but was otherwise inconsistent.

Based on these results, LAVA appears to be the most powerful and reliable tool for identifying where the causal SNP is genuinely shared between traits (in contrast to simply being situated in the same genomic region), with ρ‐HESS coming in as the second best. We also found LAVA to be the most computationally efficient and straightforward of the tools from a user perspective.

### 3.3 Colocalisation Analysis With Coloc

We examined each region of significant local correlation identified by LAVA in each of the trait pairs using LocusZoom (Pruim et al. 2010) and ruled out any where there were no significantly associated SNPs (with a cut‐off *p* < 5E‐5) in the region in either trait (Figures S5–S7). The remaining regions were then analysed in coloc, to finemap each region and determine whether the causal variants were shared between the traits (scenario H_4_) or if they represented distinct causal variants within the same region (H_3_). The results for all regions where coloc converged are shown in Table 3. Note that the LAVA regions in Table 3 with results shown on two separate lines correspond to situations where coloc identified two smaller but separate colocalising regions within the larger LAVA window.

**Table 3:** Colocalisation analysis of significant (*p* < 2E‐5) LAVA results.

Pair	LAVA region	LAVA *r*_g_	LAVA *p*	Coloc H3 PP	Coloc H4 PP	SNP trait 1	position	*p*	SNP trait 2	position	*p*	*R* ^2^	Gene
HT‐AF	Chr4:79880102‐81206182	0.761	4.28E‐08	0.0225	0.977	rs12509595	81182554	2.19e‐52	rs1458038	81164723	1.74E‐09	0.948	FGF5^a^
	Chr4:109978983‐111733579	0.388	9.41E‐10	0.999	~1E‐08	rs7685862	111389101	4.77E‐12	rs2723296	111604483	2.33E‐80	0.002	PITX1/ENPEP
	Chr7:25671576‐27351286	0.583	1.26E‐07	0.086 0.674	0.874 0.017	rs3735533 rs6961048	27245893 27328187	9.12E‐18 9.67E‐15	rs10262140 rs10262140	27256464	6.74E‐06 6.74E‐06	0.789 4.37E‐04	HOXA13^a^ EVX1/HOXA13
	Chr11:1857846‐2477449	0.575	6.29E‐06	0.0306	0.965	rs4980379	1888614	1.92E‐21	rs588321	1896957	1.88E‐06	0.824	LSP1‐TNNT3^a^
	Chr17:76596288‐77412786	0.749	1.72E‐05	0.109	0.890	rs8076588	76757296	3.01E‐07	rs7224711	76772288	3.72E‐08	0.975	CYTH1‐USP36^a^
HT‐CKD	Chr2:26894103‐28819510	0.551	3.67E‐07	0.944	0.0187	rs35021474	26916844	8.05E‐37	rs4665991	27766284	1.77E‐06	1.83E‐04	KCNK3/GCKR‐CCDC21f
	Chr4:79880102‐81206182	−0.674	2.91E‐11	0.035	0.964	rs12509595	81182554	2.19E‐52	rs1458038	81164723	4.21E‐09	0.948	FGF5^a^
	Chr16:20351262‐22667048	0.654	6.56E‐15	1.08E‐08	1	rs77924615	20392332	2.82E‐18	rs77924615	20392332	6.38E‐69	1	UMOD‐PDILT^a^
HT‐T2D	Chr1:38474037‐40200950	0.736	1.21E‐07	0.283	0.702	rs61779331	39970928	6.13E‐07	rs61779275	39820310	1.07E‐25	1	MACF1 extending HEYL, including PABPC4^a^
	Chr2:144503689‐145977920	1	2.42E‐10	0.160	0.468	rs1427298	145214421	1.49E‐06	rs12691693	145255210	1.15E‐05	0.051	ZEB2/ZEB2
	Chr3:184524269‐185709996	0.718	2.21E‐10	0.419	0.411	rs114482534	185487427	6.91E‐06	rs7633675	185510613	3.206E‐57	0.057	IGF2BP2/IGF2BP2
	Chr5:55221399‐55968966	0.561	1.21E‐07	0.040 0.007	0.959 0.992	rs458036 rs3936510	55816081 55860866	2.11E‐06 3.31E‐06	rs459193 rs9687846	55806751 55861894	6.78E‐23 3.468E‐18	0.886 0.954	Intergenic ANKRD55‐MAP3K1^a^ Intergenic ANKRD55‐MAP3K1^a^
	Chr7:130418705‐131856481	0.570	5.85E‐09	0.997	1.10E‐04	rs75672964	131321010	6.87E‐08	rs1562396	130457914	9.64E‐16	0.0023	PODXL/KLF14
	Chr8:29744541‐31134786	0.776	3.34E‐06	0.013	0.986	rs2725371	30854033	3.90E‐08	rs10954772	30863938	5.74E‐08	0.970	PURG^a^
	Chr10:114255955‐115588903	0.287	9.28E‐06	0.994 0.928	8.96E‐05 6.99E‐02	rs2479124 rs55899248	114578994 114773608	2.28E‐07 1.53E‐07	rs72824077 rs7903146	114596655 114758349	7.98E‐21 < 2.3E‐319	5.11E‐05 0.680	Intergenic/intergenic TCFL2
	Chr15:37962916‐39238840	0.654	1.01E‐05	0.430 0.248	0.015 0.432	rs10438404 rs10438404	38938017 38938017	1.04E‐05 1.04E‐05	rs34715063 rs8032939	38873115 38834033	8.40E‐15 1.88E‐08	0.082 0.053	Intergenic/intergenic intergenic/RASGRP3
	Chr16:53393883‐54866095	0.682	9.52E‐15	6.00E‐02	0.940	rs56094641	53806453	6.80E‐11	rs55872725	53809123	8.51E‐79	0.979	FTO^a^
	Chr17:45883902‐47516224	0.773	1.75E‐10	0.960 1	3.99E‐02 1.68E‐10	rs12940898 rs35073649	46981419 47422510	3.12E‐09 8.93E‐17	rs35895680 rs35895680	47060322 47060322	1.08E‐15 1.08E‐15	0.311 0.021	TTLL6‐IGF2BP1/ZNF652‐GNGT2 TTLL6‐IGF2BP1

*Note:* LAVA regions with results shown on two separate lines correspond to situations where coloc identified two smaller but separate colocalising regions within the larger LAVA window. *r*_g_ indicates the estimated local genetic correlation. Posterior probabilities for H_3_ and H_4_ as calculated by coloc are given, as well as the most significant SNP and its associated *p* value in the underlying GWAS for each trait, plus the squared correlation (*R* ^2^) between these as calculated from the 1000 Genomes European reference population. Nearest genes are given in the format gene1/gene2 when the posterior probability of H_3_ suggests that the causal SNPs are likely to be in a different genes.

Abbreviations: AF, atrial fibrillation and flutter; CKD, chronic kidney disease; HT, hypertension; T2D, type 2 diabetes.

^a^ Denotes the same causal variant is implicated as shared with both traits.

For the hypertension and atrial fibrillation pairing, signals implicating shared causal variants were seen on chromosomes 4, 7, 11, and 17, in the genes *FGF5*, *HOXA13*, in the region spanning *LSP1* to *TNNT3* and that spanning *CYTH1* to *USP36* respectively. Coloc defined two additional colocating signals (due to separate causal variants) at Chr7:25671576‐27351286 within *EVX1* and an intragenic segment that were independent of the *HOXA13* signal. A second local correlation on chromosome 4 resolved to independent causal variants in the *PITX1* and *ENPEP* genes, within 200 kb of each other. These two loci both encode transcription factors, although ENPEP has also been implicated in the upregulation of blood pressure (Yang et al. 2013) and the regulation of blood vessel development (Chen et al. 2014). LocusZoom plots of these colocalising signals are shown in Figure S5.

In hypertension and CKD, only three regions of colocalisation passed QC. These were again a signal on chromosome 4 at *FGF5*, and one on chromosome 16 for a SNP, rs77924615, within an LD block extending across *UMOD* and *PDILT*. *UMOD* is a well‐established causal gene for CKD and *FGF5* has been previously implicated in GWAS of kidney‐function‐related traits (Wuttke et al. 2019). There was also a signal on chromosome 2 that resolved into two independent signals in *KCNKR* for hypertension and *GCKR* for CKD, within 850 kb of each other. LocusZoom plots of these colocalising signals are shown in Figure S6.

It is of interest that *FGF5* lies within a colocalisation locus for not only hypertension and atrial fibrillation, but also for CKD. The protein encoded by *FGF5* belongs to the fibroblast growth factor family, which possess broad mitotic and cell survival activities, and is involved in a wide variety of processes including cell growth, morphogenesis and tissue repair. *FGF5* has been implicated in GWAS of hypertension (Ehret et al. 2011), and other fibroblast growth factors are increasingly recognised as contributors to blood pressure regulation through renal mechanisms (Tomaszewski et al. 2015). Recently it has been implicated as a therapeutic target for CKD due to its ability to mitigate inflammation (Lu and Wang 2024), which has also been demonstrated in its protective effect against liver injury by diminishing liver inflammation (Cui et al. 2022). It is likely that *FGF5* may also confer an effect in atrial fibrillation via inflammatory pathways.

Hypertension and type 2 diabetes showed the most local genetic correlation, with 10 regions highlighted by LAVA passing downstream quality control. These included 4 regions (on chromosomes 1, 5, 8, 16) where the causal variant was deemed to be shared (coloc H_4_ posterior probability > 0.7) between both traits. The chromosome 1 *MACF1*‐*HEYL* signal region includes *PABPC4* which has been implicated in type 2 diabetes through an effect mediated through visceral adipose tissue (Tang et al. 2023). Numerous studies have demonstrated that polymorphisms within the fat‐mass and obesity‐associated gene *FTO* on chromosome 16 are associated with type 2 diabetes, most likely mediated through their effect on increasing BMI (Kamura et al. 2016). In the intergenic region between *ANKRD55* and *MAP3K1* on chromosome 5, coloc identified two separate colocalising signals within the larger LAVA window that are independent of (not in LD with) each other. These mark an enhancer site (ENSR00001692010) and a CTCF binding site (ENSR00001692017) respectively; SNPs in this region have been identified as being associated with a wide range of cardio‐metabolic phenotypes (Sakaue et al. 2021).

There were also several instances of independent (not a shared causal variant) signals (coloc H_3_ posterior probability > 0.7) within the same gene, for example at *PODXL*/*KLF14* on chromosome 7 and *IGF2BP1* on chromosome 17. Both *IGF2BP1* on chromosome 17 and *IGF2BP2* on chromosome 3 (for which the coloc posterior probability is shared equally between H_3_ and H_4_) are insulin growth factor binding proteins, and, along with *FTO* and *PABPC4*, are enriched within gene ontology sets regulating the metabolism and stabilisation of mRNA (GO:0061013, GO:1903311, GO:0006401, GO:0070934.) Gene set overlaps were identified in the Molecular Signature Database (https://www.gsea-msigdb.org/gsea/msigdb/human/annotate.jsp) (Subramanian et al. 2005).

LocusZoom plots of these colocalising signals for hypertension and type 2 diabetes are shown in Figure S7. Due to the long and complex LD blocks in the MHC region, it was not possible to adequately finemap the signals detected by LAVA in this region with coloc.

### 3.4 SNP‐Based Screening Approach

Our approach used thus far employed local correlation analysis, followed by colocalisation analysis with coloc, to identify potential shared genetic causes of disease. An arguably simpler “first‐pass” approach might be to scan across the genome, searching for windows (genomic segments) that contain genome‐wide significant signals in both (or all) traits of interest, with the resulting genomic segments then taken forward for analysis with coloc. Given that our primary question of interest was to identify *shared* causal variants, we used the LAVA default partitions (which were designed to minimize LD between blocks) as a starting point and searched for association signals in the real data sets that met genome‐wide significance (5E‐08) for both traits and where the top associated variants (one for each trait) lay within 500 kb of one another. This generated an additional 44 regions (28 for type 2 diabetes and hypertension, 11 for atrial fibrillation and hypertension, and 5 for CKD and hypertension), that had not previously been identified using LAVA, where a follow‐up analysis using coloc was applied.

The coloc results are shown in Table S5. The vast majority of the loci appeared to involve separate causal variants (coloc H3 posterior probability > 0.7); only seven instances where the causal variant was deemed to be shared between both traits (coloc H4 posterior probability > 0.7) were seen. Interestingly six of these seven loci had been identified using LOGODetect, suggesting that LOGODetect's scan statistic approach may have some conceptual links with this simpler SNP‐based screening approach.

### 3.5 Analysis Using HDL‐L

Finally, we repeated our genome‐wide local correlation analysis of the real and simulated traits using the newly‐developed HDL‐L package (Li et al. 2025). The developers of HDL‐L had previously found that HDL‐L demonstrated superior performance over LAVA in terms of computational efficiency, estimation accuracy and statistical robustness, generally detecting many fewer significant loci (and thus presumably many fewer false positives) than LAVA.

Table 4 shows all statistically significant regions (*p* < 0.05/2468 = 2.03E‐05, based on a Bonferroni correction for the 2468 HDL‐L defined test loci) obtained from HDL‐L analysis of the real and simulated traits. Consistent with previous observations (Li et al. 2025), HDL‐L identified fewer significant loci than LAVA when applied to the real traits. Specifically, ten significant regions were found: one for CKD and hypertension and nine for type 2 diabetes and hypertension. Seven of these ten regions had previously been identified by LAVA and one had been identified by LOGODetect and SNP‐based screening. Of the two completely novel findings, one was not taken forward to colocalisation analysis on account of having no significantly associated SNPs with *p* < 5E‐5 in type 2 diabetes, while the other was taken forward and generated a coloc H_4_ posterior probability of 0.964 (Figure S8), suggesting the presence of a shared causal variant at this locus. For the simulated traits, HDL‐L was successful at identifying all four true shared signals and did not detect any significant local correlation in the regions where the causal variants were unique to the individual traits, even when they were situated close together, in the same genomic region.

**Table 4:** All statistically significant regions (*p* < 2.03E‐05) from HDL‐L analysis of the real and simulated traits. *r*_g_ indicates the estimated local genetic correlation.

Trait1	Trait2	Chr	Start	End	*r*_g_	*r*_g_ lower bound	*r*_g_ upper bound	*p*	Notes
CKD	HT	16	20351262	22667048	0.934	0.713	1.000	1.32E‐06	Also identified by LAVA (*p* = 6.56E‐15). Coloc H4 PP = 1.000 (see Table 3)
T2D	HT	3	11997659	12859209	1.000	0.784	1.000	9.84E‐06	Also identified by LAVA (*p* = 4.18E‐09). Top HT *p* = 9.39E‐05 failed the threshold for taking forward to coloc.
T2D	HT	5	55221399	55968966	0.920	0.646	1.000	1.57E‐06	Also identified by LAVA (*p* = 1.21E‐07). Coloc H4 PP = 0.959 and 0.992 (see Table 3)
T2D	HT	6	42103739	43770626	1.000	0.747	1.000	7.60E‐06	Also identified by LOGODetect (*p* = 1.99E‐04) and SNP‐based screening. LAVA *p* = 0.00035. Coloc H4 PP = 0.985 (see Table S5)
T2D	HT	6	49528165	50940767	0.937	0.651	1.000	2.00E‐05	LAVA *p* = 0.00016. Coloc H4 PP = 0.964 (see Figure S8)
T2D	HT	6	163336362	164687462	1.000	0.751	1.000	1.13E‐05	Also identified by LAVA (*p* = 5.68E‐06). Coloc did not converge.
T2D	HT	7	130418705	131856481	0.801	0.504	0.980	1.34E‐05	Also identified by LAVA (*p* = 5.85E‐09). Coloc H4 PP = 1.10E‐04 (see Table 3)
T2D	HT	12	1942427	3069506	1.000	0.700	1.000	7.54E‐06	LAVA *p* = 0.00037. Top T2D *p* = 0.00022 fails threshold for taking forward to coloc.
T2D	HT	14	102004378	103617472	1.000	0.846	1.000	1.34E‐06	Also identified by LAVA (*p* = 1.57E‐07). Top HT *p* = 7.20E‐05 failed the threshold for taking forward to coloc.
T2D	HT	16	53393883	54866095	0.917	0.686	1.000	6.06E‐08	Also identified by LAVA (*p* = 9.52E‐15). Coloc H4 PP = 0.940 (see Table 3)
Trait A	Trait B	1	75132093	76097247	−1.000	−1.000	−0.854	3.41E‐07	True shared signal
Trait A	Trait B	10	14820756	15946677	0.991	0.950	1.000	4.20E‐22	True shared signal
Trait A	Trait C	1	75132093	76097247	0.989	0.942	1.000	4.27E‐13	True shared signal in close proximity to two HDL‐L windows
Trait A	Trait C	1	76097248	76977915	1.000	0.887	1.000	3.31E‐08	True shared signal in close proximity to two HDL‐L windows
Trait A	Trait C	2	134359060	135160197	−0.989	−1.000	−0.869	3.33E‐10	True shared signal in close proximity to two HDL‐L windows
Trait A	Trait C	2	135160198	137061003	−1.000	−1.000	−0.876	1.01E‐07	True shared signal in close proximity to two HDL‐L windows
Trait B	Trait C	1	75132093	76097247	−0.988	−1.000	−0.782	1.69E‐06	True shared signal
Trait B	Trait C	12	119301062	120567740	1.000	0.929	1.000	3.55E‐13	True shared signal

## 4 Discussion

Here we investigated the extent to which local genetic correlation analysis, as implemented in five recently‐developed software packages, might provide useful insight into the shared genetic basis between traits or conditions of interest, focussing on three pairings of traits that are of particular relevance to the ADMISSION study of MLTC in hospital settings. Our findings were complicated by the lack of concordance seen between the different tools when applied to the same input data; following further investigation through computer simulations, we concluded that LAVA was generally the most powerful and reliable tool for identifying when the causal SNP is shared between traits, as well as being the most computationally efficient and user‐friendly of the tools, although the newly‐developed tool HDL‐L was arguably equally user‐friendly and computationally efficient, while potentially offering better control of type 1 error. With respect to the signals seen for the real traits, visualisation through LocusZoom plots and colocalisation analysis using the coloc program confirmed that LAVA had indeed successfully identified a number of regions where genetic signals were shared between the traits; notably, in 11 instances, coloc suggested that these corresponded to implication of the same underlying causal variant.

We found that the differences in results generated by LAVA, ρ‐HESS and SUPERGNOVA could not be attributed purely to their different partitioning of the genome into test regions or segments. Remaining differences might perhaps partly be attributed to the theoretical differences between the three approaches between the packages previously described (Werme et al. 2022) (namely that (a) unlike LAVA, both ρ‐HESS and SUPERGNOVA make no specific accommodations for binary phenotypes; (b) ρ‐HESS and LAVA use different numbers of principal components when inverting the externally‐estimated LD matrix; (c) ρ‐HESS constructs its test statistics assuming that the sampling distributions of the local genetic correlation and covariance estimates are normal, while LAVA uses a simulation‐based approach to directly generate *p*‐values; and (d) SUPERGNOVA takes a random‐effects perspective—in contrast to the fixed‐effects perspective of ρ‐HESS and LAVA—and thus ends up estimating a fundamentally different quantity, with a different interpretation, from that estimated by ρ‐HESS and LAVA). LOGODetect could also be considered to use a fundamentally different (scan statistic based) approach from the other tools, perhaps partly explaining the lack of concordance between the results from LOGODetect and those from the other tools.

Local genetic correlation measures the extent to which the contribution of the SNPs in the test region to the overall genetic values (breeding values) of an individual, for separate two traits, are correlated. As pointed out by the authors of ρ‐HESS (Shi et al. 2017), this is a slightly different quantity to the correlation of the actual causal effects operating in the region (which measures the extent to which the effect of a SNP—the beta coefficient in a regression model—on one trait is correlated with its effect on the other trait). Only in the special case where there is no LD between SNPs will the genetic covariance and the covariance of causal effects coincide. LAVA, ρ‐HESS and SUPERGNOVA all make attempts to remove the effects of LD through decorrelation, but they use slightly different strategies to achieve this goal.

Alternative approaches to modelling pleiotropy (the phenomenon whereby a genetic variant has an effect on multiple traits) include multi‐trait analysis of genome‐wide association summary statistics using software such as MTAG (Turley et al. 2018) or MultiPhen (O'Reilly et al. 2012), or meta‐analysis‐based approaches such as CCMA (Baurecht et al. 2016) or ASSET (Bhattacharjee et al. 2012). These approaches typically analyse single variants, one at a time, and are often focussed on improving the power to detect an association with one (or more) traits by taking into account the association with the other trait(s), rather than on determining whether the traits share the same genetic cause. A more direct approach to addressing the question of whether two or more traits share the same underlying causal variants is to use colocalisation analysis (Giambartolomei et al. 2014; Giambartolomei et al. 2018), as was performed here for the regions that had been identified through local correlation analysis in LAVA. However, colocalisation analysis is computationally prohibitive to carry out on a genome‐wide scale, and so is not well suited as a “first‐pass” analysis for detection of potential shared genomic regions. An arguably simpler “first‐pass” approach is to scan across the genome, searching for windows (genomic segments) that contain genome‐wide significant signals in both (or all) traits of interest; our investigation of this approach suggested that it primarily detects regions where the traits are governed by separate causal variants, although we did find a few instances where shared causal variants were implicated, most of which were also detected using LOGODetect. It is possible that reducing the maximum distance allowed between the top association signals would lead to a higher rate of identification of shared causal variants, while also reducing the number of regions to take forward for colocalisation analysis. The LocusZoom plots for the signals implicated through our real data analyses (Figures S5–S, 7) suggest that LAVA is, however, rather successful at identifying such colocalising signals. While LAVA enjoys a principled theoretical justification for the test statistic that it generates, it has been shown to be more prone to false inference than its recently‐developed competitor, HDL‐L. Perhaps this is not too important if one is planning to take forward any regions identified for further analysis and verification (e.g., through colocalisation analysis). However, limiting the number of regions to take forward to those that are identified by HDL‐L, and are thus less likely to be false positives, certainly has some appeal.

It is perhaps debatable whether the regions of genetic correlation identified—and thus potentially implicated genes—in our analyses of hypertension together with atrial fibrillation and flutter, hypertension together with CKD, and hypertension together with diabetes, offer any important new biological insight in comparison to that gleaned from previous GWAS of the individual conditions. Hypertension, atrial fibrillation, CKD, and diabetes have all been extensively studied previously via GWAS and follow‐up (post‐GWAS) analysis, and much biological insight into contributing mechanisms and pathways underlying these conditions has already been obtained. However, the fact that local correlation analysis, particularly as implemented in LAVA, was able to highlight a number of shared genetic regions that were further corroborated through colocalisation analysis provides a proof‐of‐principle for adopting the use of this approach more widely, particularly in application to other, less well‐studied, conditions. Of note, the identification of both the *UMOD* locus and *FGF5* in relation to hypertension and CKD using local correlation as implemented in LAVA, backed up in both cases by coloc as being attributable to a shared causal variant, establishes the potential utility of this method to identify potential shared mechanisms of disease; interestingly, both *UMOD* and *FGF5* are currently being explored as therapeutic targets for these conditions.

Limitations of the work carried out here include the fact that summary statistics from large‐scale GWAS, the starting point for all the methods implemented here, are inherently prone to potential misspecifications and biases (Privé et al. 2022), including but not limited to those attributable to heterogeneity in phenotype definition. In our own real data analysis, we restricted ourselves to considering only three specific pairs of traits, all of which included hypertension, primarily on account of their prior identification as being of interest within the context of MLTC in hospital settings (Robertson et al. 2022). This contrasts with the more extensive sets of traits considered, mostly for illustrative purposes, by the developers of the packages themselves (namely 36 complex traits and diseases for ρ‐HESS, 30 complex traits for SUPERGNOVA, 7 neuropsychiatric traits for LOGODetect, 25 behavioural and health‐related traits for LAVA, and 30 phenotypes for HDL‐L). Our own results concerning the lack of concordance seen between the different tools might, therefore, not be generally applicable across other conditions, although we note that our conclusions are, to a large extent, backed up by the results seen in the simulated data sets. Our simulation study itself could be considered somewhat simplistic; it certainly does not mimic the degree of complexity and polygenicity expected to be operating in most common, complex, disorders (Wray et al. 2018). However, it does capture the main relevant aspects in terms of modelling genetic factors that operate on multiple conditions along with both clustered and dispersed genetic factors that operate only on a single condition.

## Author Contributions

**Rebecca Darlay:** formal analysis, investigation, writing – original draft, review and editing. **Rupal L. Shah:** formal analysis, investigation, writing – review and editing. **Richard M. Dodds:** conceptualization, funding acquisition, supervision. **Anand T. N. Nair:** investigation, writing – review and editing. **Ewan R. Pearson:** conceptualization, funding acquisition, supervision, writing – review and editing. **Miles D. Witham:** conceptualization, funding acquisition, supervision, writing – review and editing. **Heather J. Cordell:** conceptualization, funding acquisition, supervision, writing – original draft, review and editing.

## Conflicts of Interest

The authors declare no conflicts of interest.

## Supporting information

Supporting Figure S1: Regions of local correlation as detected by LAVA (top panel), ρ‐HESS (middle panel) and SUPERGNOVA (bottom panel) under different genomic partition definitions.

Supporting Figure S2: A comparison of regions of local correlation as detected by LAVA, ρ‐HESS and SUPERGNOVA when using either the LAVA default partitions (top panel), the ρ‐HESS default partitions (middle panel) or the SUPERGNOVA default partitions (bottom panel).

Supporting Figure S3: Exploration of LAVA analysis of hypertension and type 2 diabetes on chromosomes 1 and 5 using different partition definitions.

Supporting Figure S4: A LocusZoom plot demonstrating the LD in the chr2 region simulated to be significantly associated with traits B and C.

Supporting Figure S5: LocusZoom plots, LAVA and coloc results of significantly associated locally correlated regions between hypertension and atrial fibrillation, as detected by LAVA.

Supporting Figure S6: LocusZoom plots, LAVA and coloc results of significantly associated locally correlated regions between hypertension and CKD, as detected by LAVA.

Supporting Figure S7: LocusZoom plots, LAVA and coloc results of significantly associated locally correlated regions between hypertension and type 2 diabetes, as detected by LAVA.

Supporting Figure S8: LocusZoom plot and coloc results for new locus identified by HDL‐L.

Supporting Table S1: Listing of the GWAS summary statistics used and their sample sizes.

Supporting Table S2: Exploration of results from LAVA analysis of hypertension and type 2 diabetes in selected regions using different partition definitions.

Supporting Table S3: Results of local correlation analysis of the simulated traits in the regions where each trait pair shares two identical causal SNPs. r_g_ indicates the estimated local genetic correlation.

Supporting Table S4: Results of local correlation analysis of the simulated traits in the regions where there is a causal SNP in the same region for two traits, but those causal SNPs are in no or very low LD with each other. r_g_ indicates the estimated local genetic correlation. *R* ^2^ indicates the correlation between the relevant causal SNPs, as calculated by PLINK in the 1000 Genomes European reference population.

Supporting Table S5: Coloc results for regions identified via a SNP‐based screening approach. Results shown on separate lines correspond to situations where coloc identified several smaller but separate colocalising regions within the larger window. Results where the causal variant was deemed to be shared between the traits (coloc H4 posterior probability PP.H4 > 0.7) are marked in bold.

ADMISSION Research Collaborative (29th July 2024).
