# Supplementary figures and images for: Exploring Similarities and Differences Between Methods That Exploit Patterns of Local Genetic Correlation to Identify Shared Causal Loci Through Application to Genome‐Wide Association Studies of Multiple Long Term Conditions
Source: Genet Epidemiol. 2025 Jun 19;49(5):e70012. doi: 10.1002/gepi.70012 (PMC12179580; doi:10.1002/gepi.70012)

## LAVA

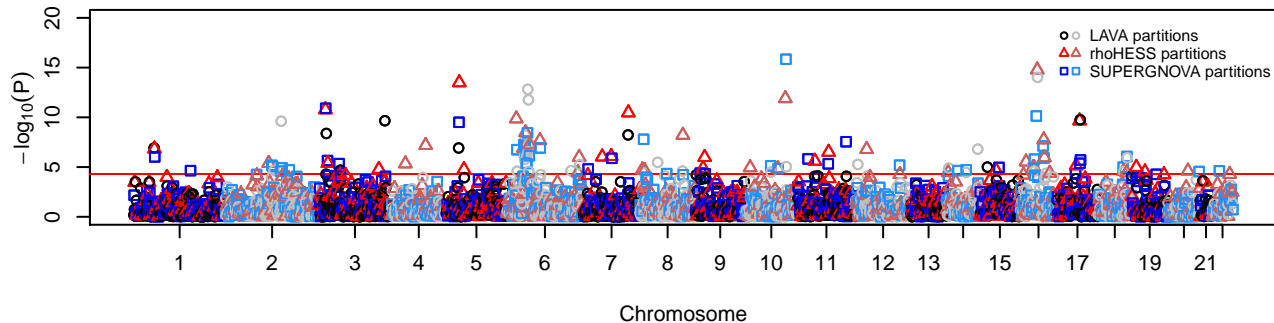

## rhoHES

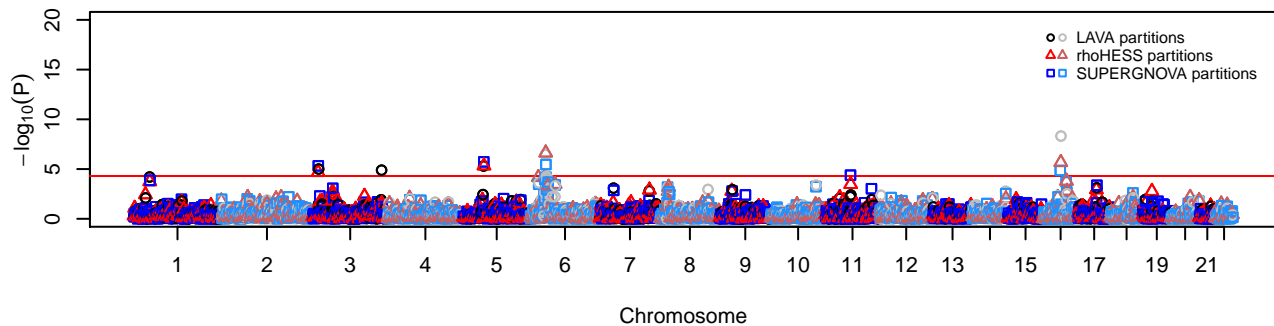

## SUPERGNOVA

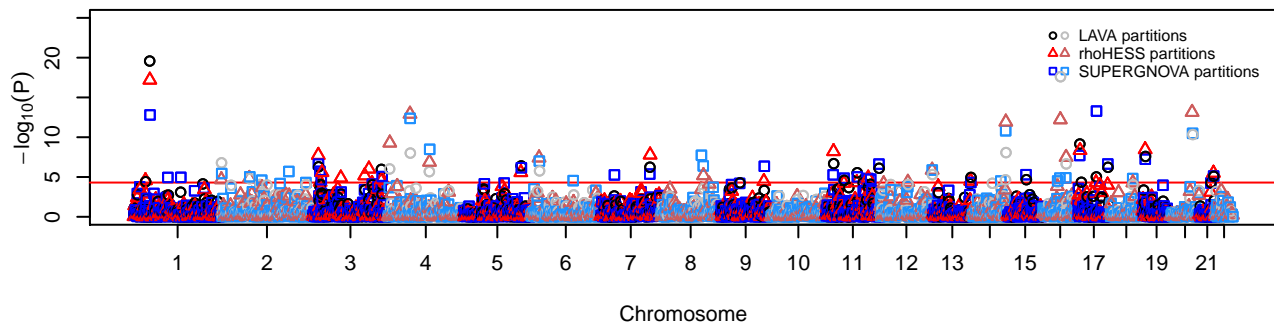

Supplement: Supplementary file 1 — Supporting Figure S1: Regions of local correlation as detected by LAVA (top panel), ρ‐HESS (middle panel) and SUPERGNOVA (bottom panel) under different genomic partition definitions. [file GEPI-49-0-s007.pdf]

### LAVA Partitions

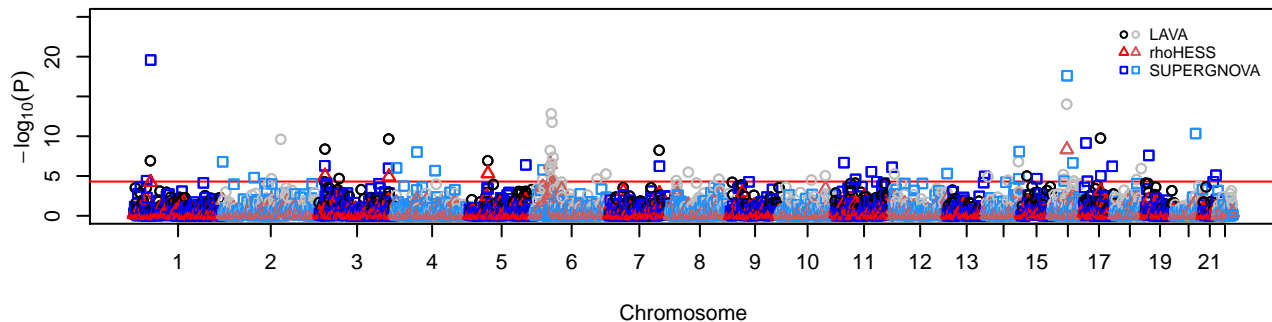

### rhoHESS Partitions

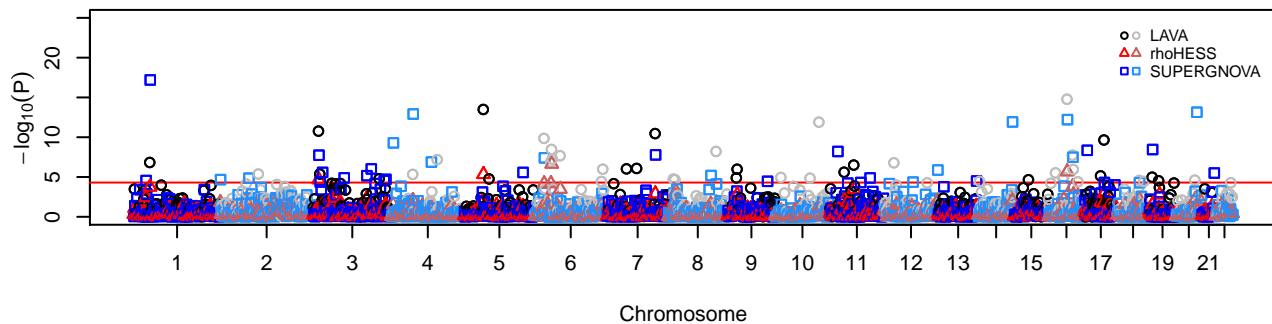

### SUPERGNOVA Partitions

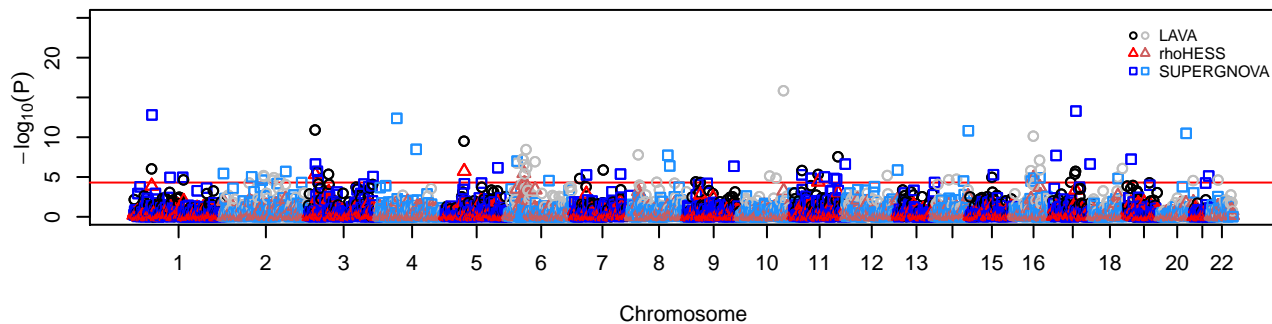

Supplement: Supplementary file 2 — Supporting Figure S2: A comparison of regions of local correlation as detected by LAVA, ρ‐HESS and SUPERGNOVA when using either the LAVA default partitions (top panel), the ρ‐HESS default partitions (middle panel) or the SUPERGNOVA default partitions (bottom panel). [file GEPI-49-0-s014.pdf]

A. Chromosome 1 region

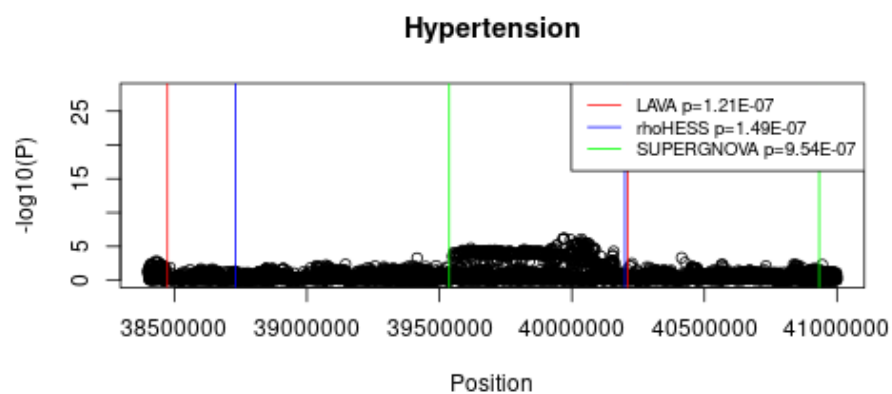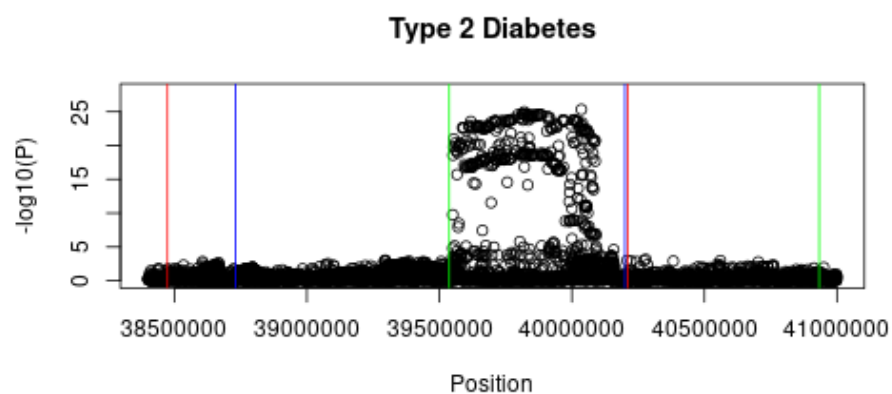

B. Chromosome 5 region

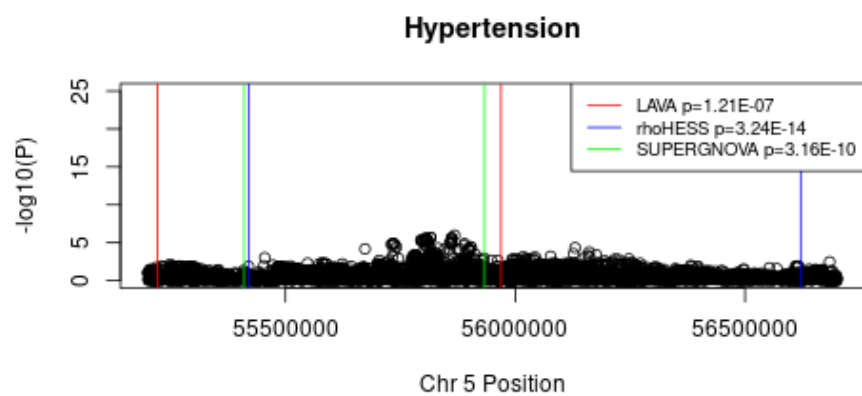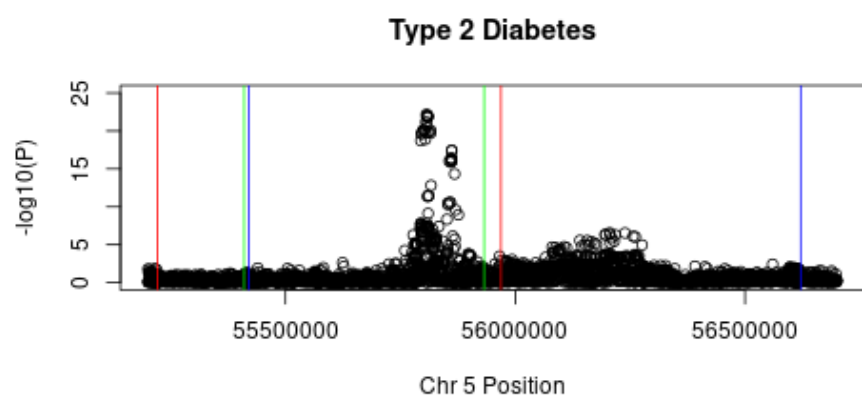

Supplement: Supplementary file 3 — Supporting Figure S3: Exploration of LAVA analysis of hypertension and type 2 diabetes on chromosomes 1 and 5 using different partition definitions. [file GEPI-49-0-s010.pdf]

**Trait B**

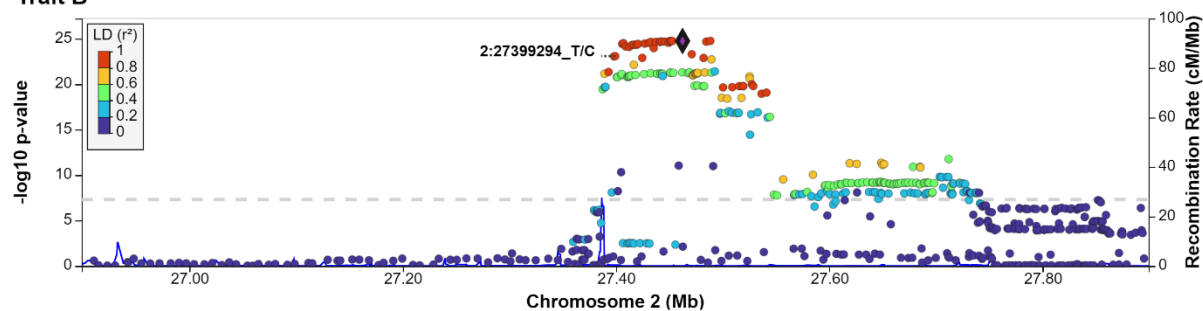

**Trait C**

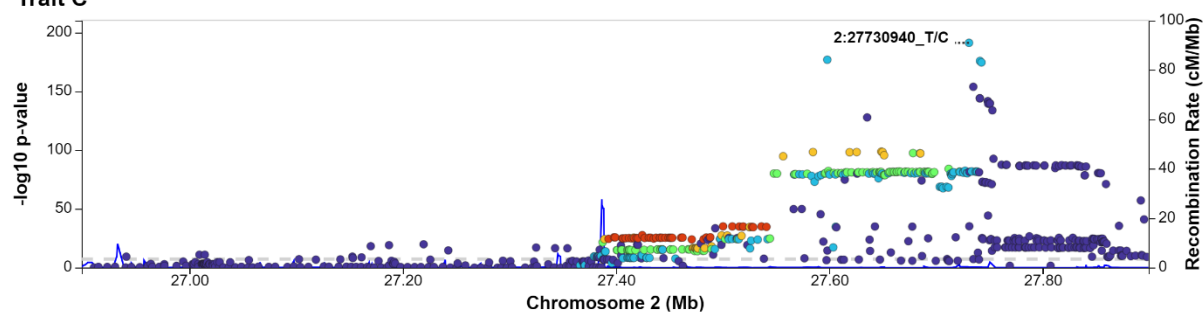

**GWAS Catalog hits for Trait C**

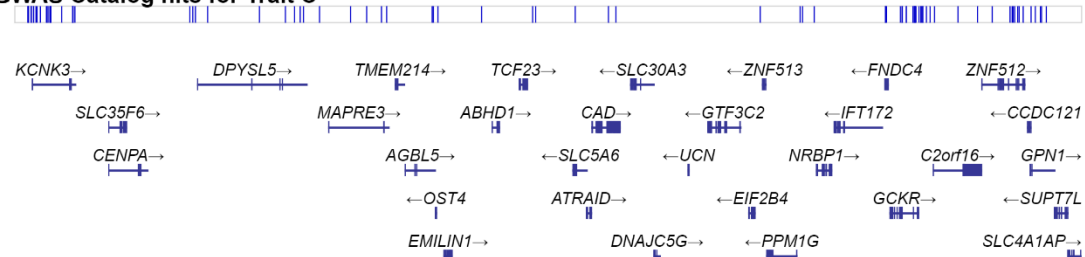

Supplement: Supplementary file 4 — Supporting Figure S4: A LocusZoom plot demonstrating the LD in the chr2 region simulated to be significantly associated with traits B and C. [file GEPI-49-0-s008.pdf]
