# Supplementary material for: Exploring Similarities and Differences Between Methods That Exploit Patterns of Local Genetic Correlation to Identify Shared Causal Loci Through Application to Genome‐Wide Association Studies of Multiple Long Term Conditions
Source: Genet Epidemiol. 2025 Jun 19;49(5):e70012. doi: 10.1002/gepi.70012 (PMC12179580; doi:10.1002/gepi.70012)
Supplement: Supplementary file 5 — Supporting Figure S5: LocusZoom plots, LAVA and coloc results of significantly associated locally correlated regions between hypertension and atrial fibrillation, as detected by LAVA. [file GEPI-49-0-s012.pdf]

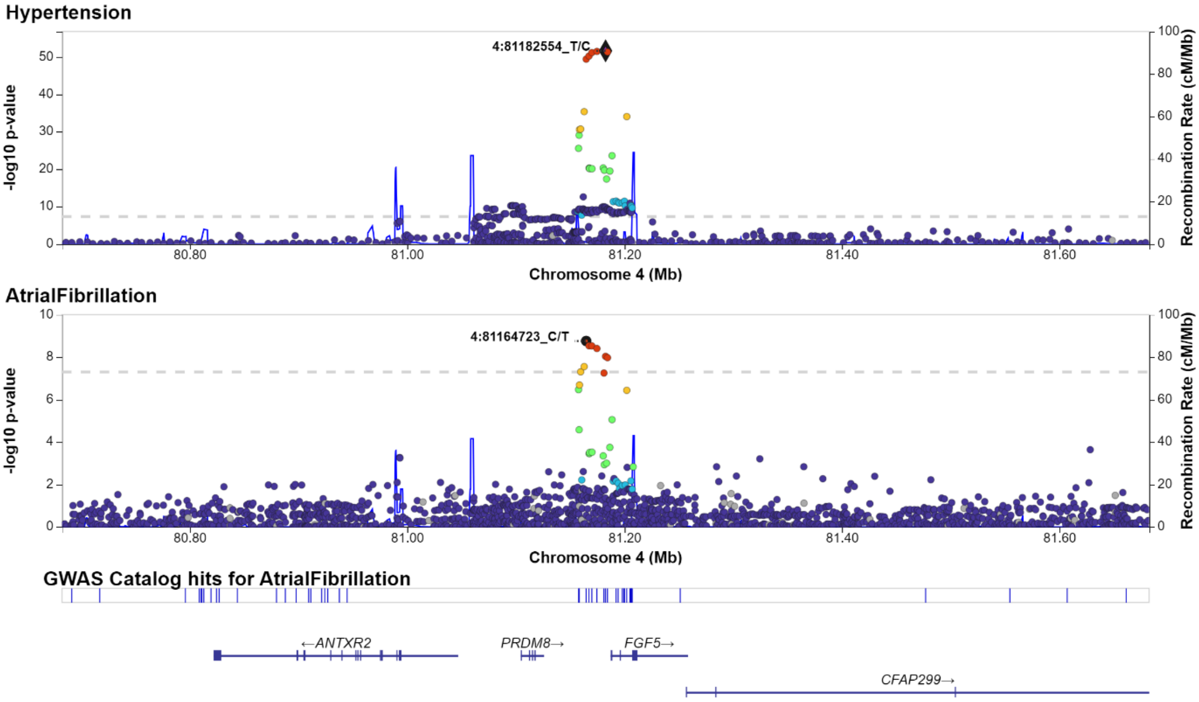

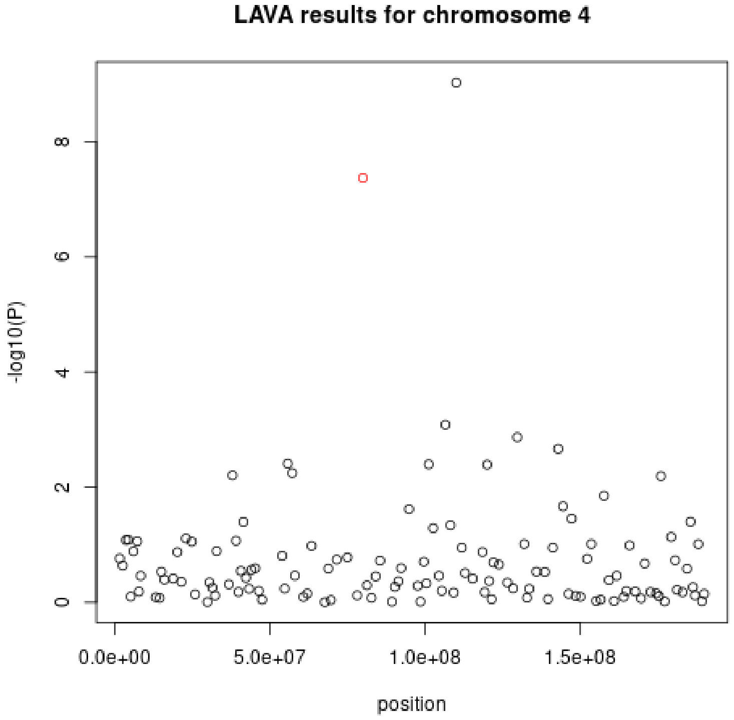

LAVA region	LAVA r_g	LAVA P	Coloc H3 PP	Coloc H4 PP	SNP HT	position	GWAS_P	SNP AF	position	GWAS_P
Chr4:79880102-81206182	0.761	4.28E-08	0.0225	0.977	rs12509595	81182554	2 .19e-52	rs1458038	81164723	1.74E-09

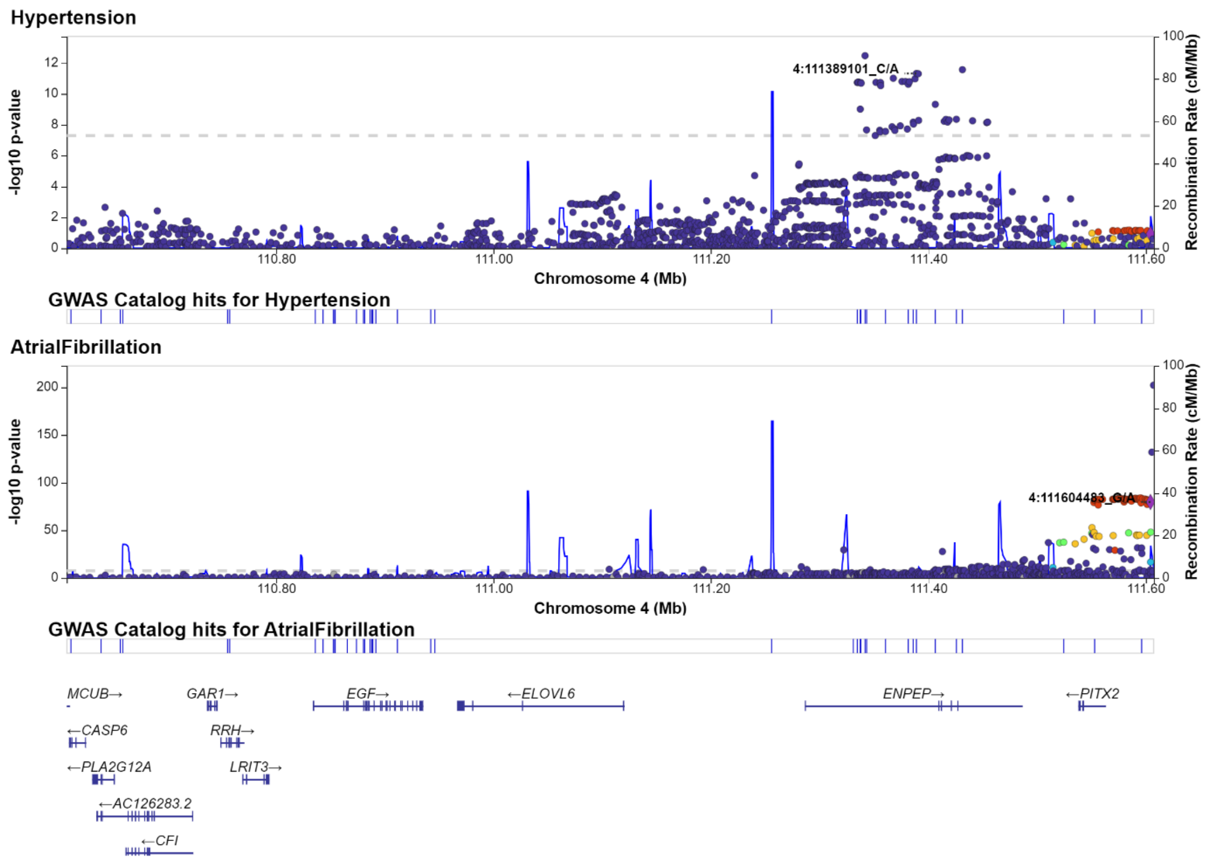

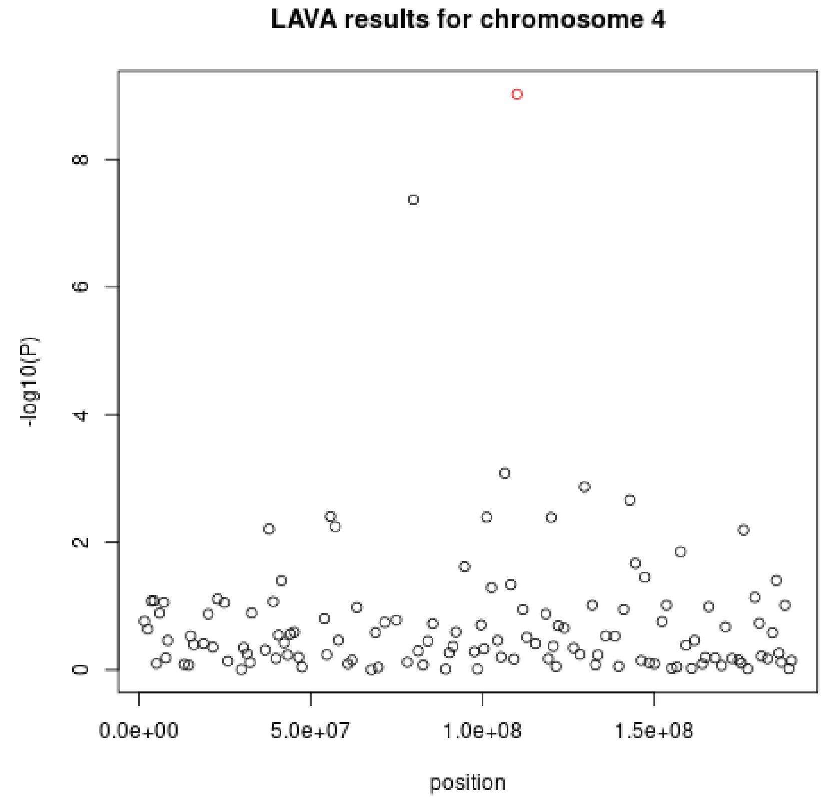

LAVA region	LAVA r_g	LAVA P	Coloc H3 PP	Coloc H4 PP	SNP HT	position	P	SNP AF	position	P
Chr4:109978983-111733579	0.388	9.41E-10	0.999	~1e-08	rs7685862	111389101	4.77E-12	rs2723296	111604483	2.33E-80

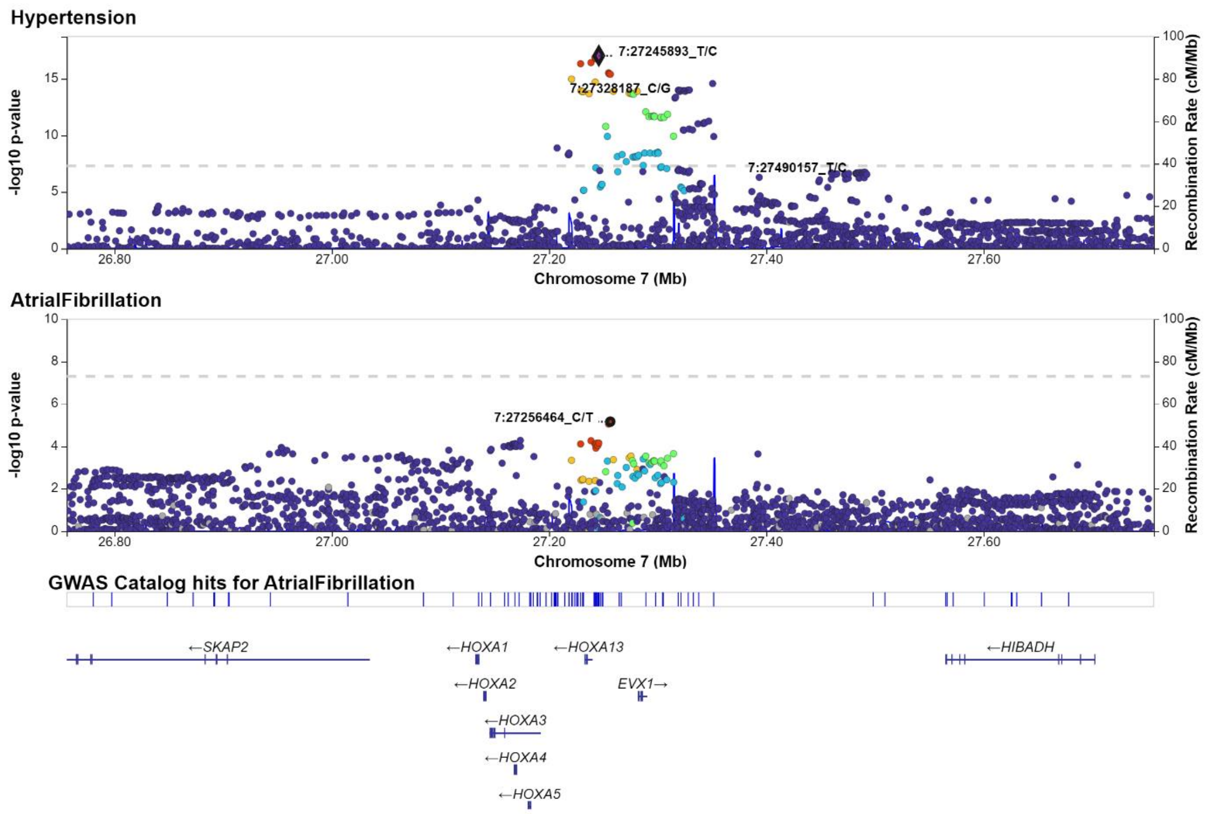

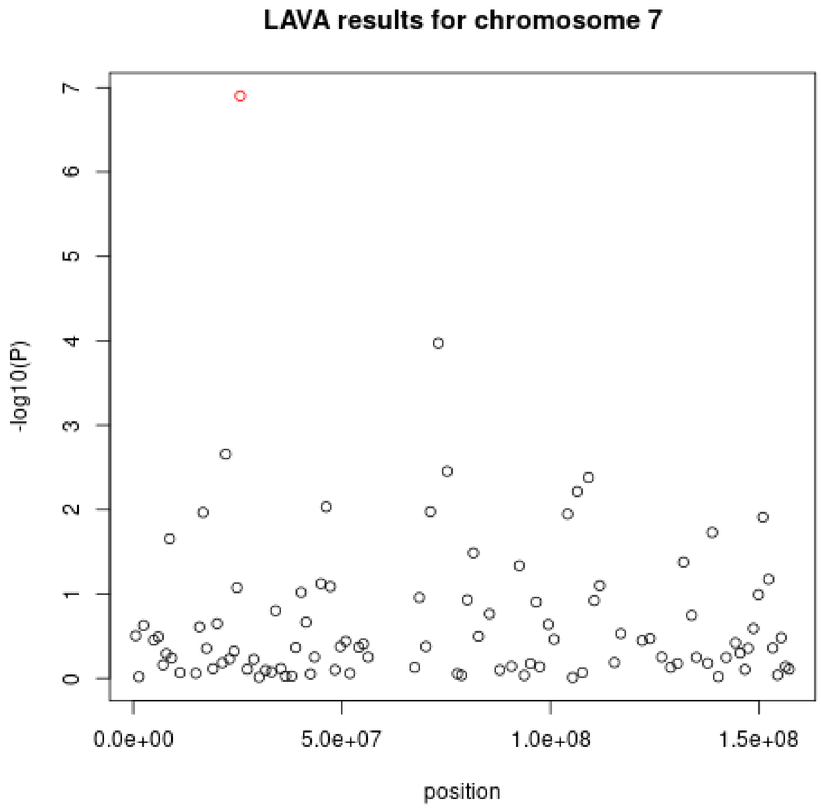

LAVA region	LAVA r_g	LAVA P	Coloc H3 PP	Coloc H4 PP	SNP HT	position	P	SNP AF	position	P
Chr7:25671576-27351286	0.583	1.26E-07	0.086<br>0.674	0.874<br>0.017	rs3735533<br>rs6961048	27245893<br>27328187	9.12E-18<br>9.67E-15	rs10262140<br>rs10262140	27256464	6.74E-06<br>6.74E-06

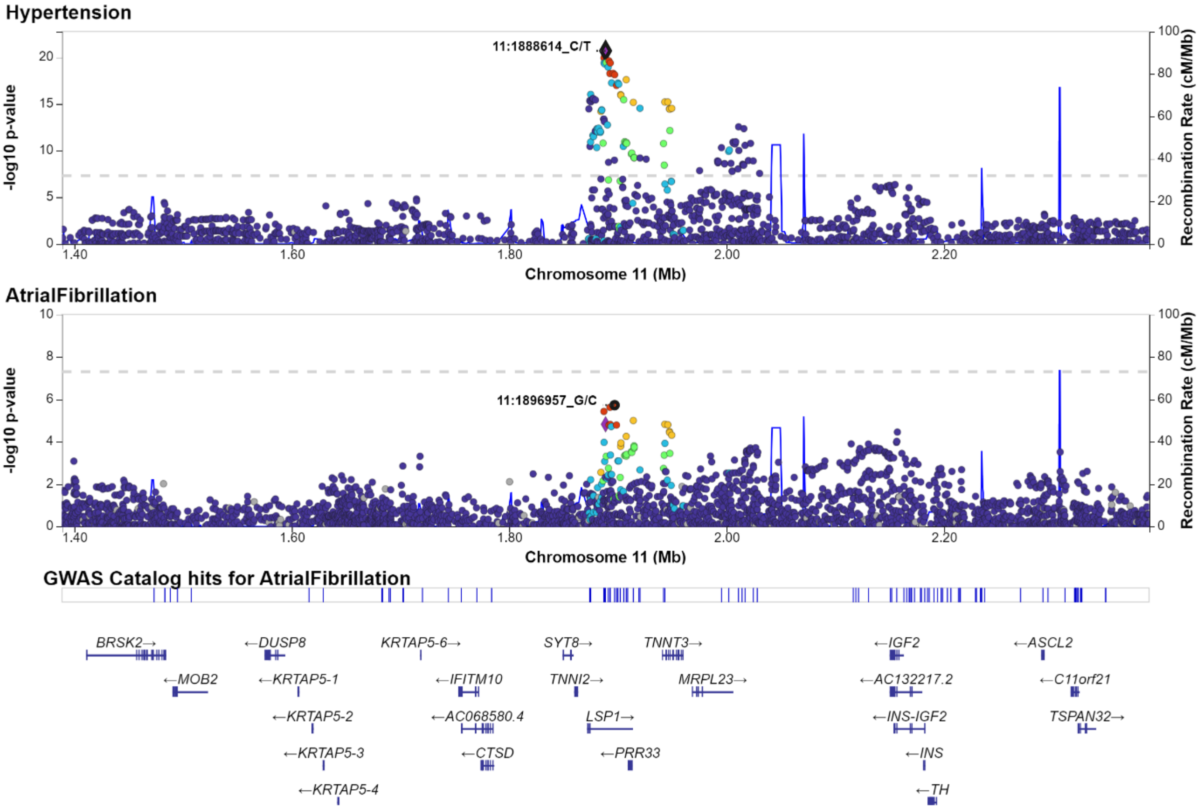

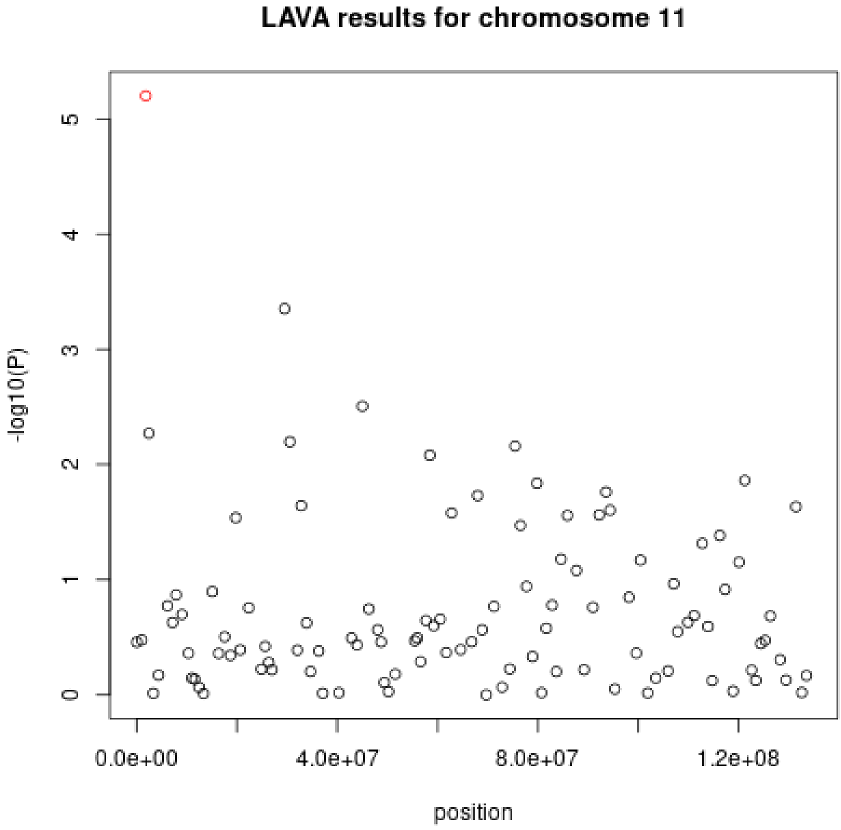

LAVA region	LAVA r_g	LAVA P	Coloc H3 PP	Coloc H4 PP	SNP HT	position	P	SNP AF	position	P
Chr11:1857846-2477449	0.575	6.29E-06	0.0306	0.965	rs4980379	1888614	1.92E-21	rs588321	1896957	1.88E-06

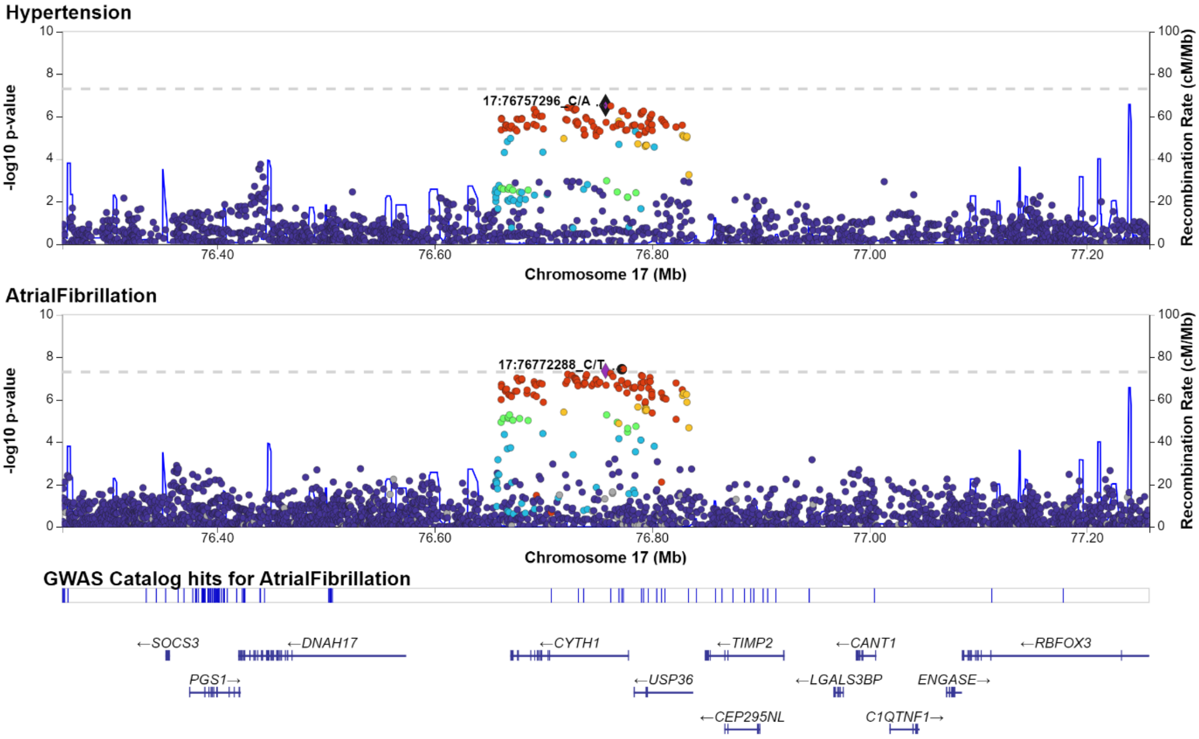

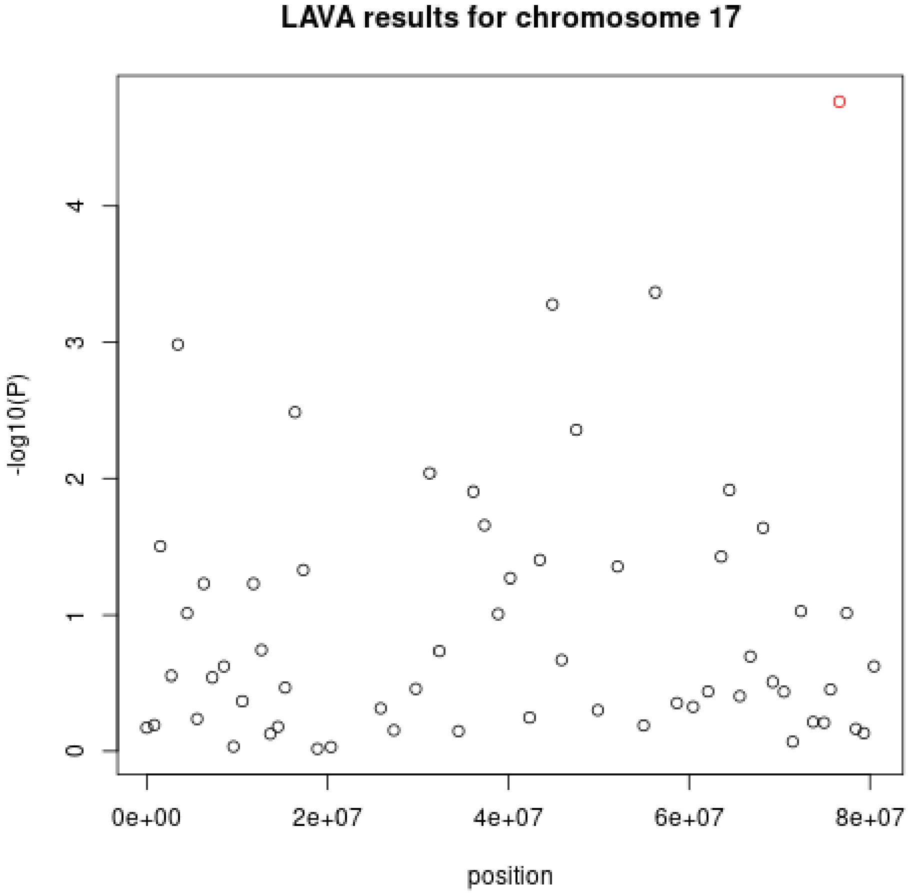

LAVA region	LAVA r_g	LAVA P	Coloc H3 PP	Coloc H4 PP	SNP HT	position	P	SNP AF	position	P
Chr17:76596288-77412786	0.749	1.72E-05	0.109	0.890	rs8076588	76757296	3.01E-07	rs7224711	76772288	3.72E-08
