# Supplementary material for: Exploring Similarities and Differences Between Methods That Exploit Patterns of Local Genetic Correlation to Identify Shared Causal Loci Through Application to Genome‐Wide Association Studies of Multiple Long Term Conditions
Source: Genet Epidemiol. 2025 Jun 19;49(5):e70012. doi: 10.1002/gepi.70012 (PMC12179580; doi:10.1002/gepi.70012)
Supplement: Supplementary file 6 — Supporting Figure S6: LocusZoom plots, LAVA and coloc results of significantly associated locally correlated regions between hypertension and CKD, as detected by LAVA. [file GEPI-49-0-s013.pdf]

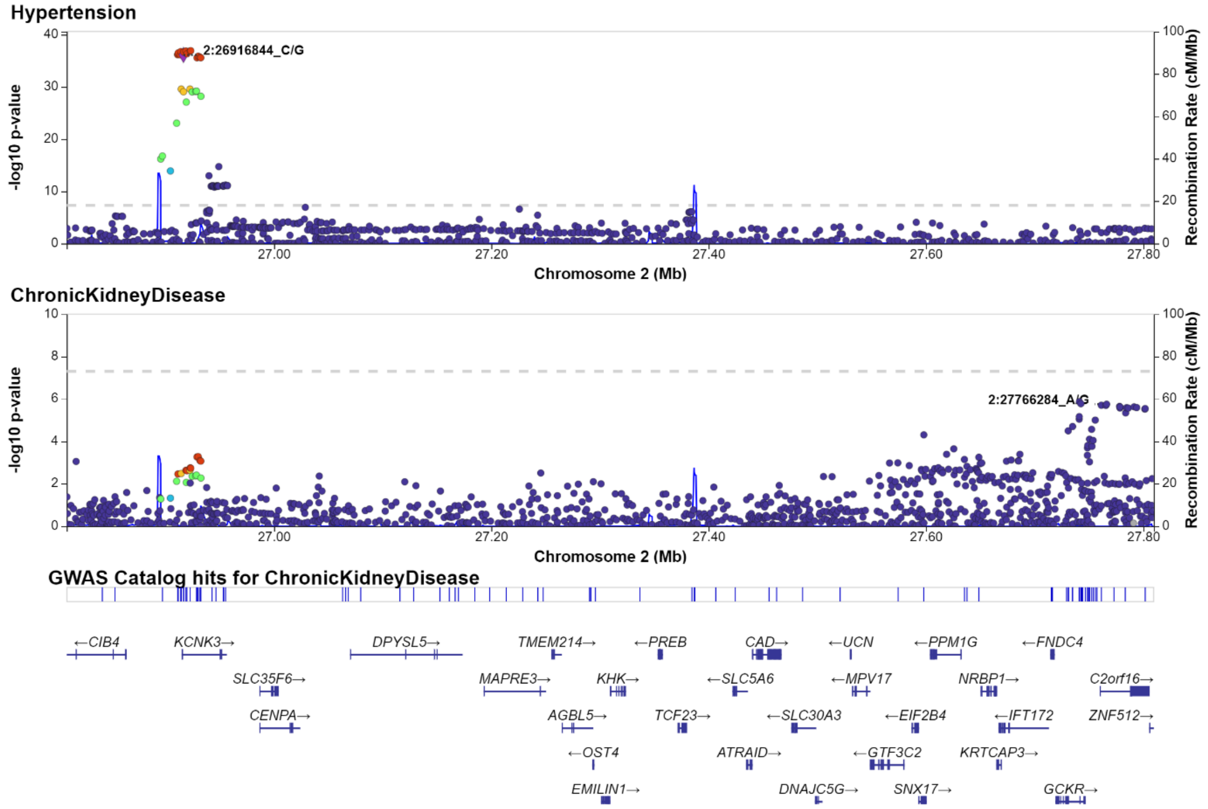

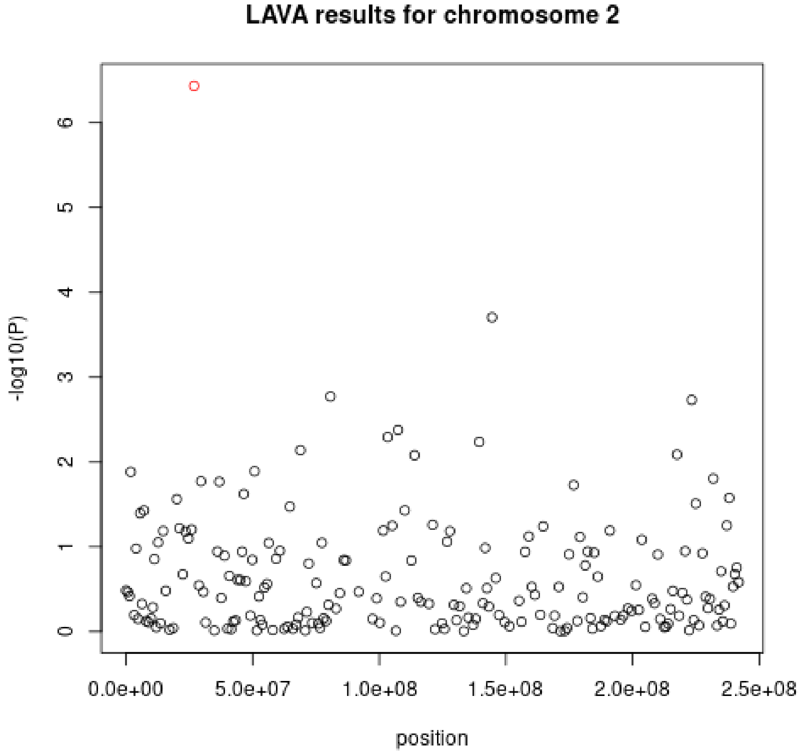

LAVA region	LAVA r_g	LAVA P	Coloc H3 PP	Coloc H4 PP	SNP HT	position	GWAS_P	SNP CKD	position	GWAS_P
Chr2:26894103-28819510	0.551	3.67E-07	0.944	0.0187	rs35021474	26916844	8.05E-37	rs4665991	27766284	1.77E-06

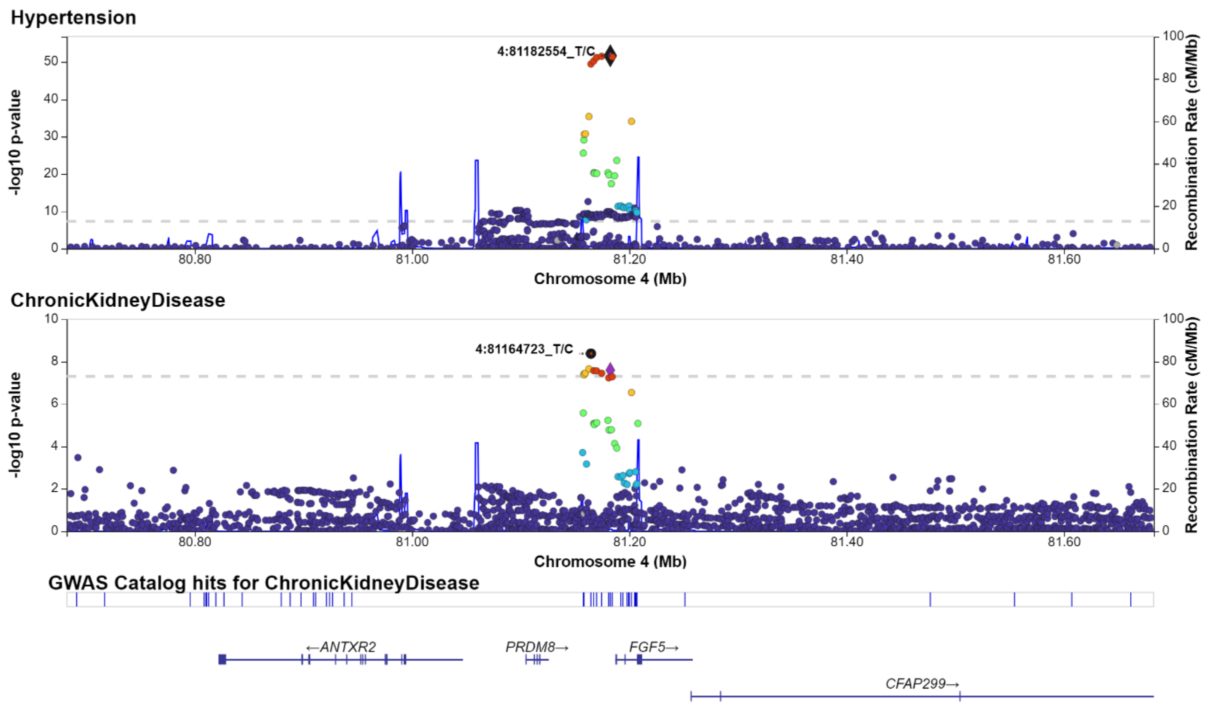

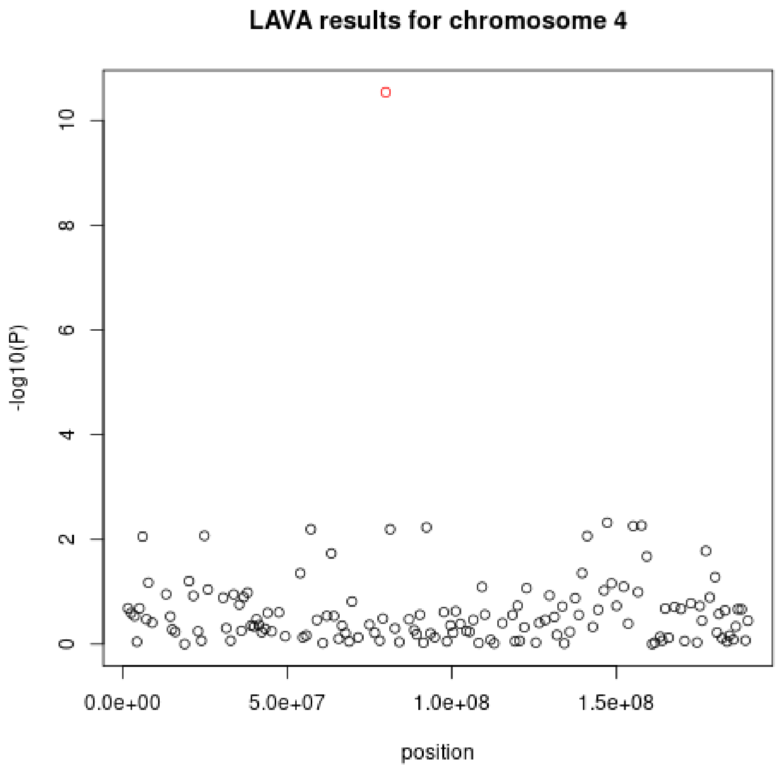

LAVA region	LAVA r_g	LAVA P	Coloc H3 PP	Coloc H4 PP	SNP HT	position	GWAS_P	SNP CKD	position	GWAS_P
Chr4:79880102-81206182	-0.674	2.91E-11	0.035	0.964	rs12509595	81182554	2.19E-52	rs1458038	81164723	4.21E-09

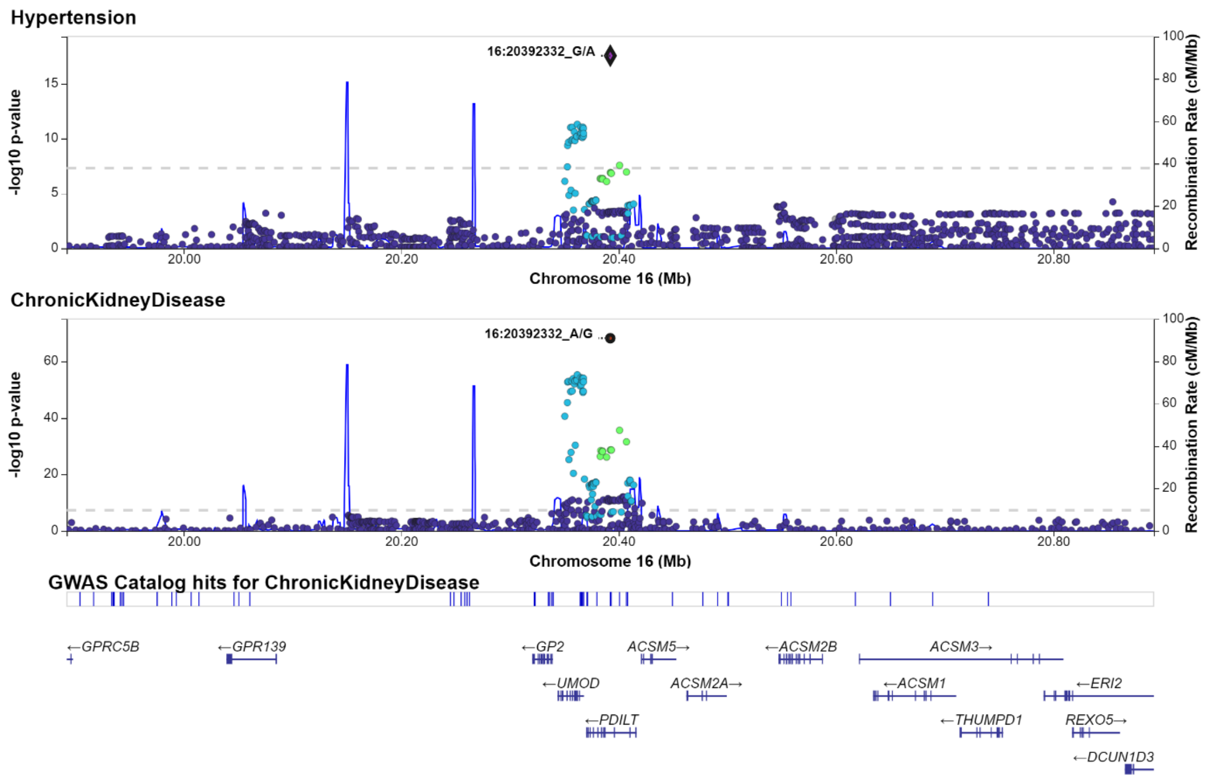

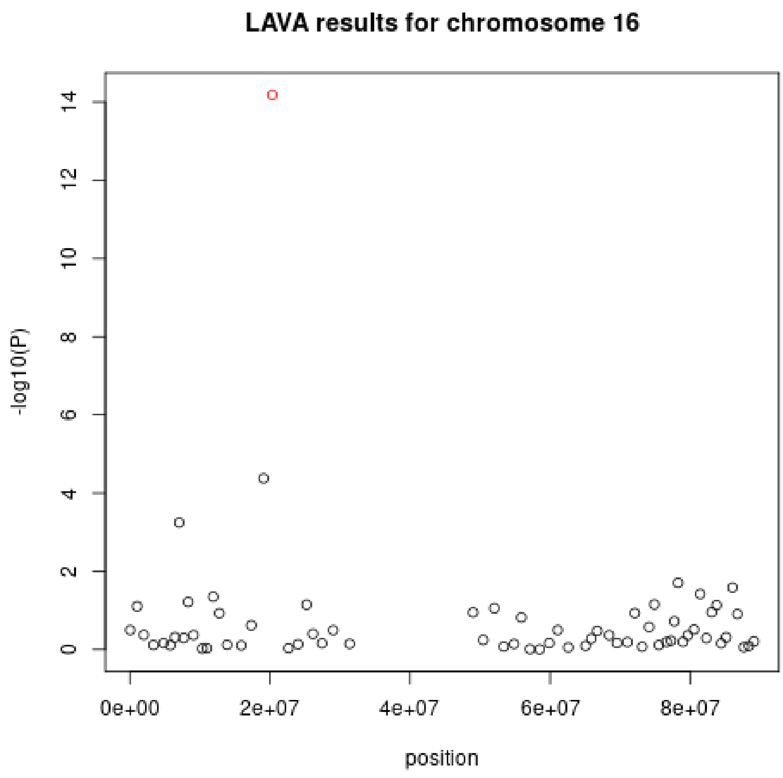

LAVA region	LAVA r_g	LAVA P	Coloc H3 PP	Coloc H4 PP	SNP HT	position	GWAS_P	SNP CKD	position	GWAS_P
Chr16:20351262-22667048	0.654	6.56E-15	1.08E-08	1	rs77924615	20392332	2.82E-18	rs77924615	20392332	6.38E-69
