# Supplementary material for: Exploring Similarities and Differences Between Methods That Exploit Patterns of Local Genetic Correlation to Identify Shared Causal Loci Through Application to Genome‐Wide Association Studies of Multiple Long Term Conditions
Source: Genet Epidemiol. 2025 Jun 19;49(5):e70012. doi: 10.1002/gepi.70012 (PMC12179580; doi:10.1002/gepi.70012)
Supplement: Supplementary file 7 — Supporting Figure S7: LocusZoom plots, LAVA and coloc results of significantly associated locally correlated regions between hypertension and type 2 diabetes, as detected by LAVA. [file GEPI-49-0-s005.pdf]

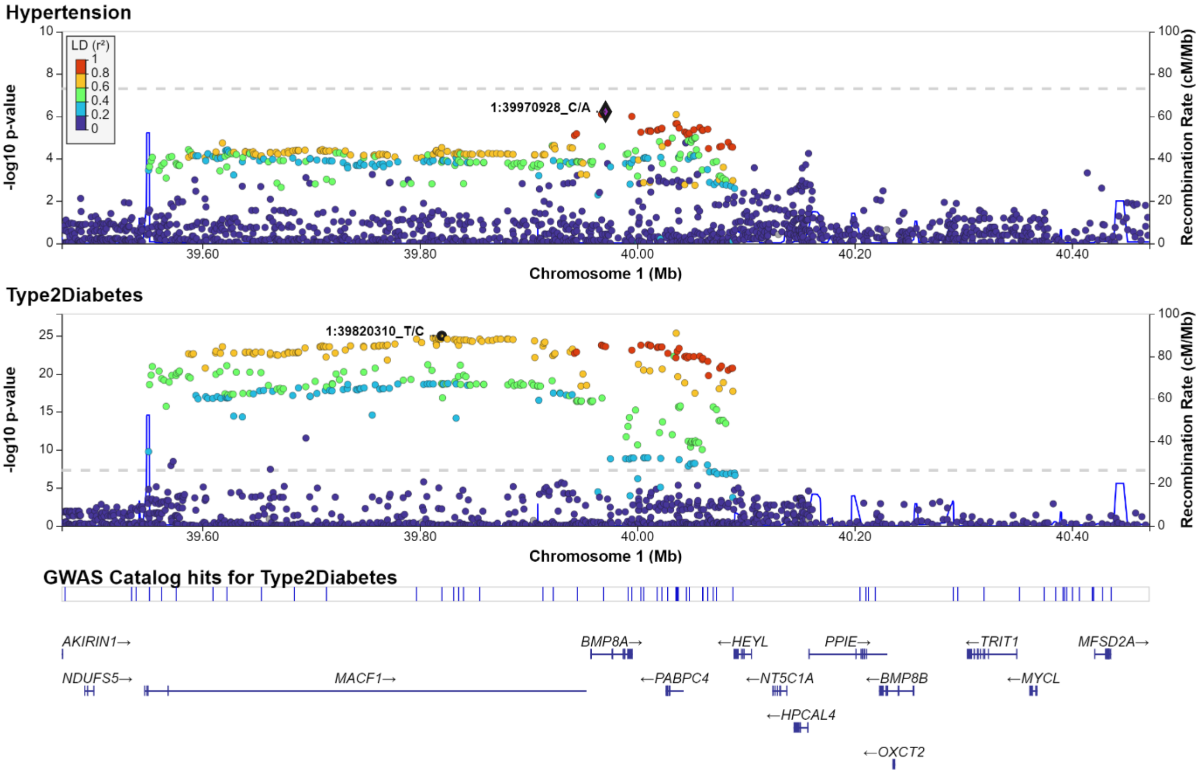

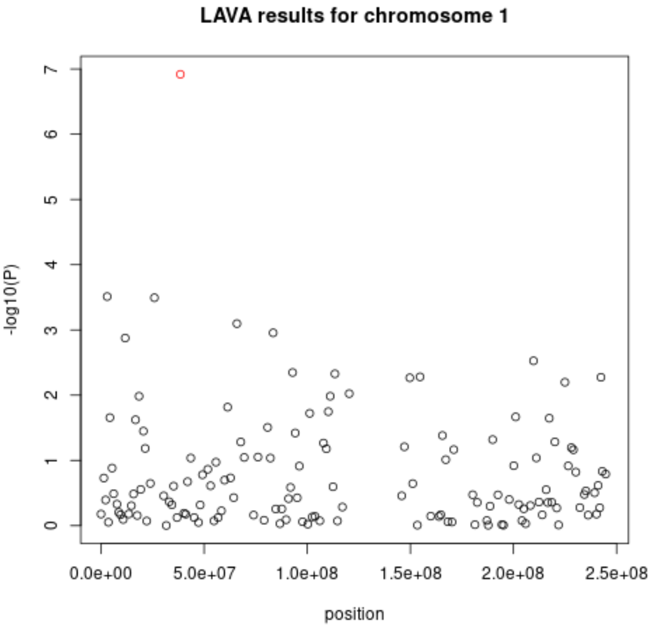

LAVA region	LAVA r_g	LAVA P	Coloc H3 PP	Coloc H4 PP	SNP Trait 1	position	P	SNP trait 2	position	P
Chr1:38474037-40200950	0.736	1.21E-07	0.283	0.702	rs61779331	39970928	6.13E-07	rs61779275	39820310	1.07E-25

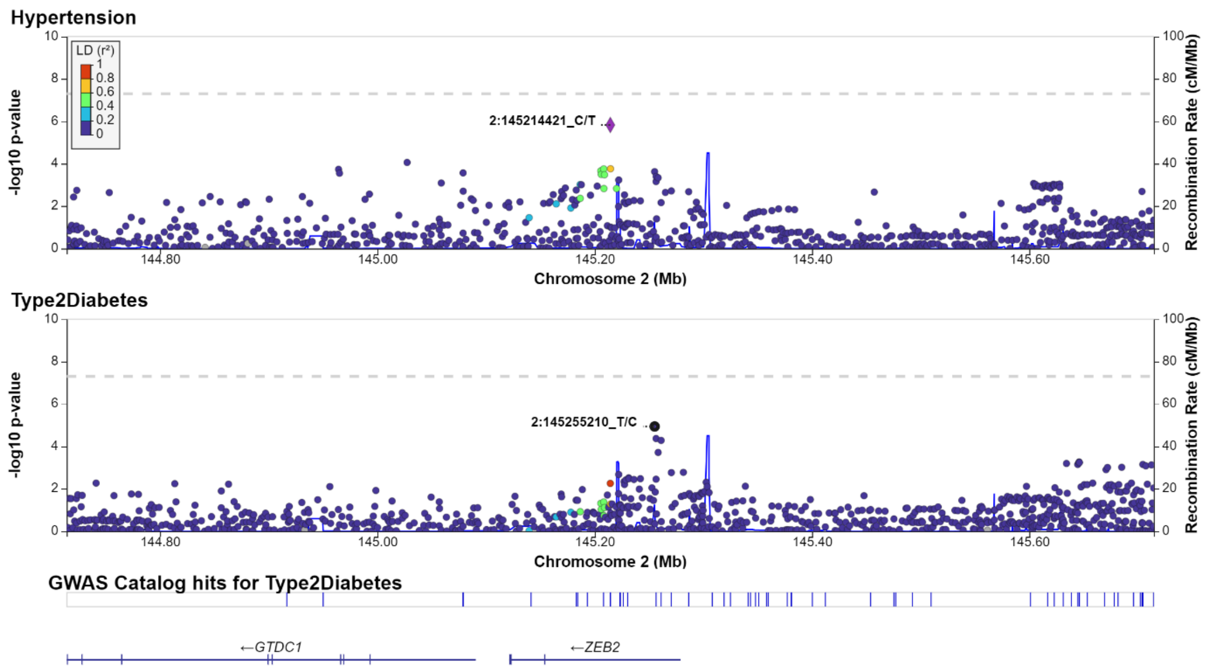

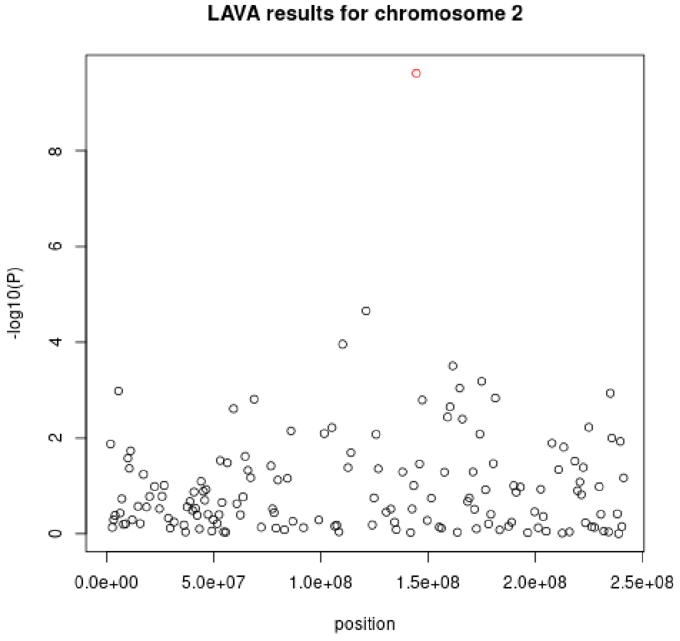

LAVA region	LAVA r_g	LAVA P	Coloc H3 PP	Coloc H4 PP	SNP Trait 1	position	P	SNP trait 2	position	P
Chr2:144503689-145977920	1	2.42E-10	0.160	0.468	rs1427298	145214421	1.49E-06	rs12691693	145255210	1.15E-05

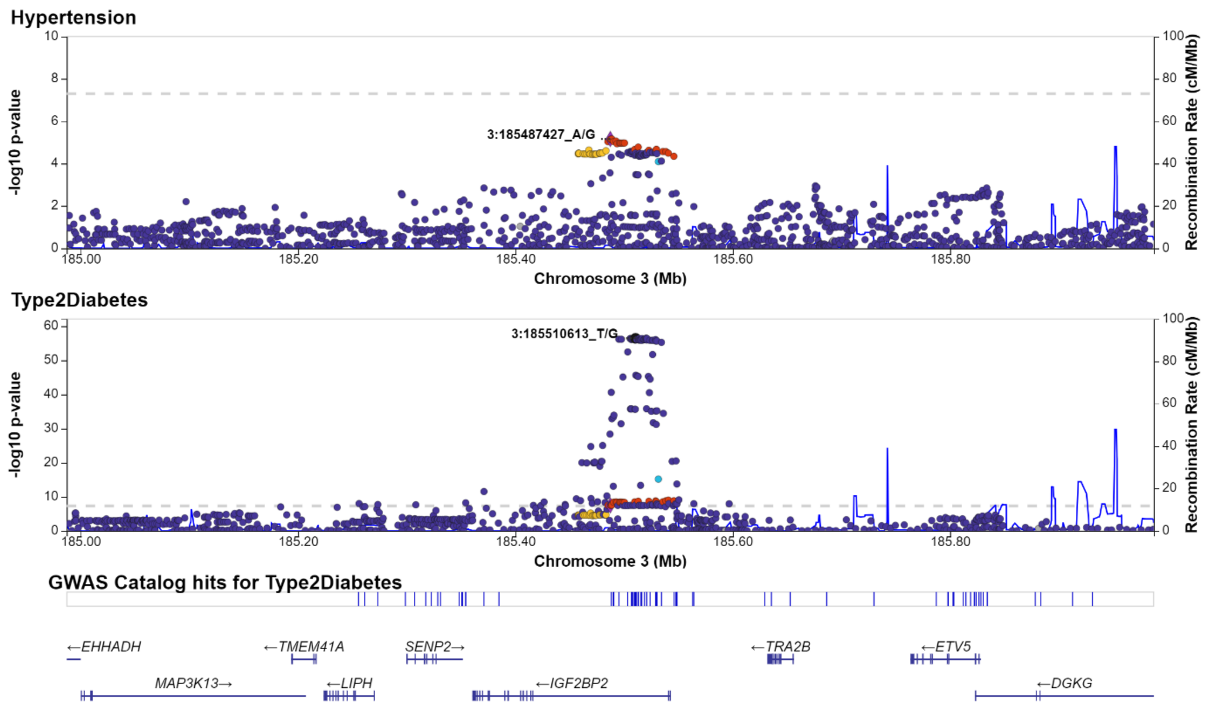

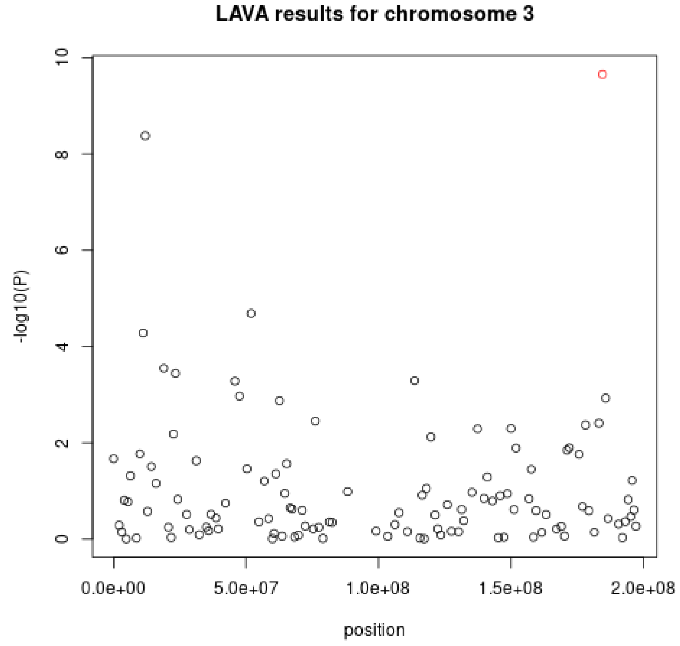

LAVA region	LAVA r_g	LAVA P	Coloc H3 PP	Coloc H4 PP	SNP Trait 1	position	P	SNP trait 2	position	P
Chr3:184524269-185709996	0.718	2.21E-10	0.419	0.411	rs114482534	185487427	6.91E-06	rs7633675	185510613	3.206E-57

*coloc could not analyse LAVA signal in window 11997659-12859209 (p = 4.18E-09) as the underlying signal in the hypertension data (rs2347100 p = 9.3859e-05) was below its significance threshold to identify a colocalising signal

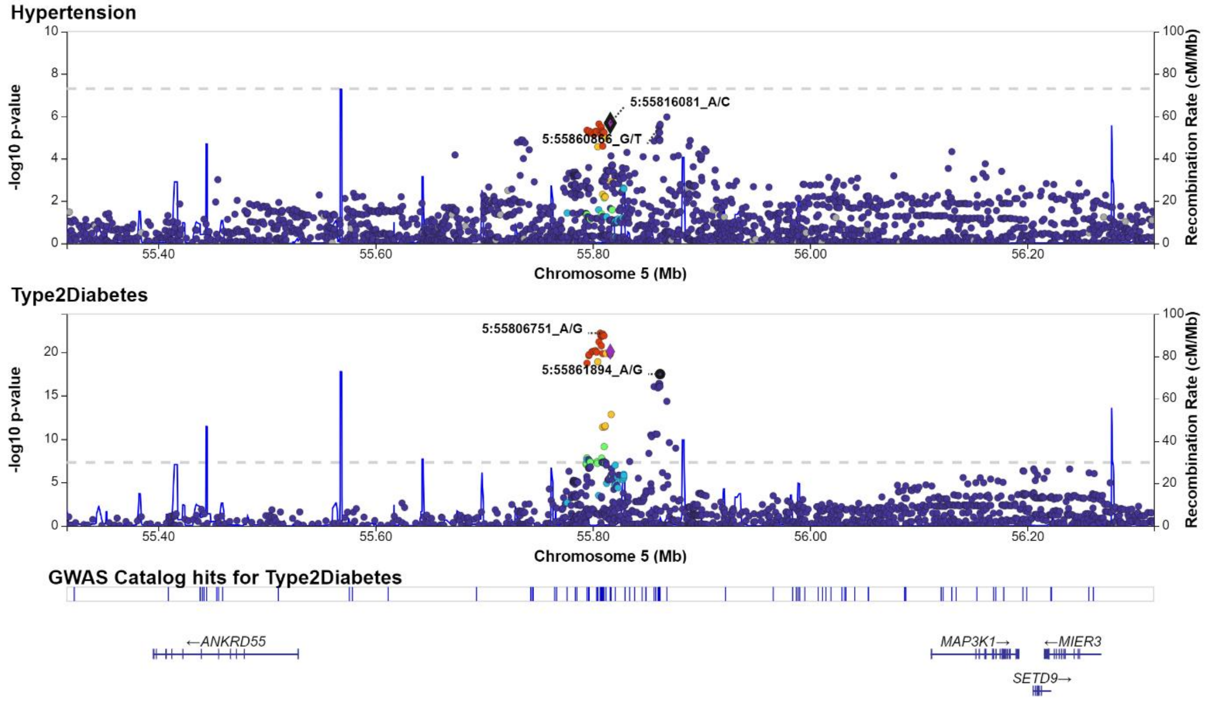

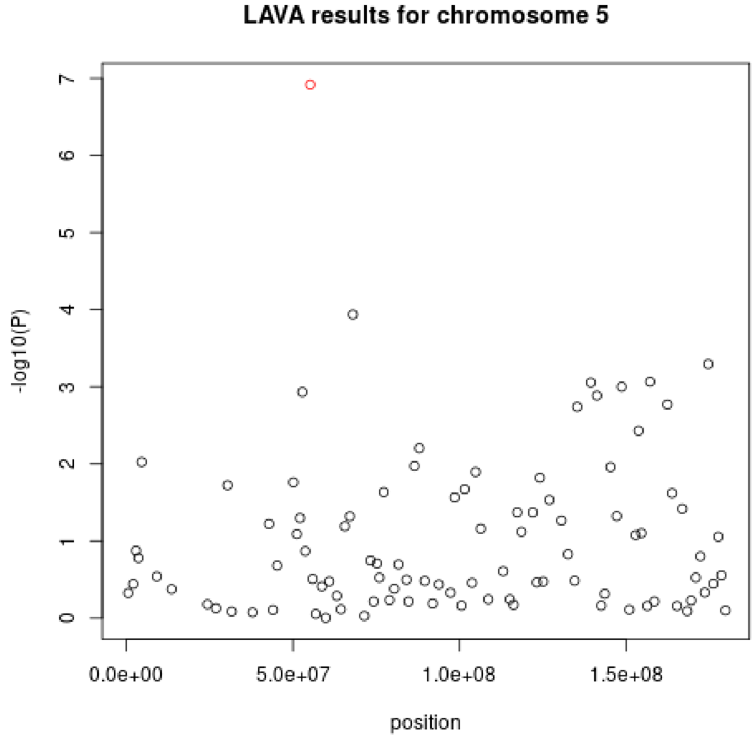

LAVA region	LAVA r_g	LAVA P	Coloc H3 PP	Coloc H4 PP	SNP Trait 1	position	P	SNP trait 2	position	P
Chr5:55221399-55968966	0.561	1.21E-07	0.040	0.959	rs458036	55816081	2.11E-06	rs459193	55806751	6.78E-23
			0.007	0.992	rs3936510	55860866	3.31E-06	rs9687846	55861894	3.468E-18

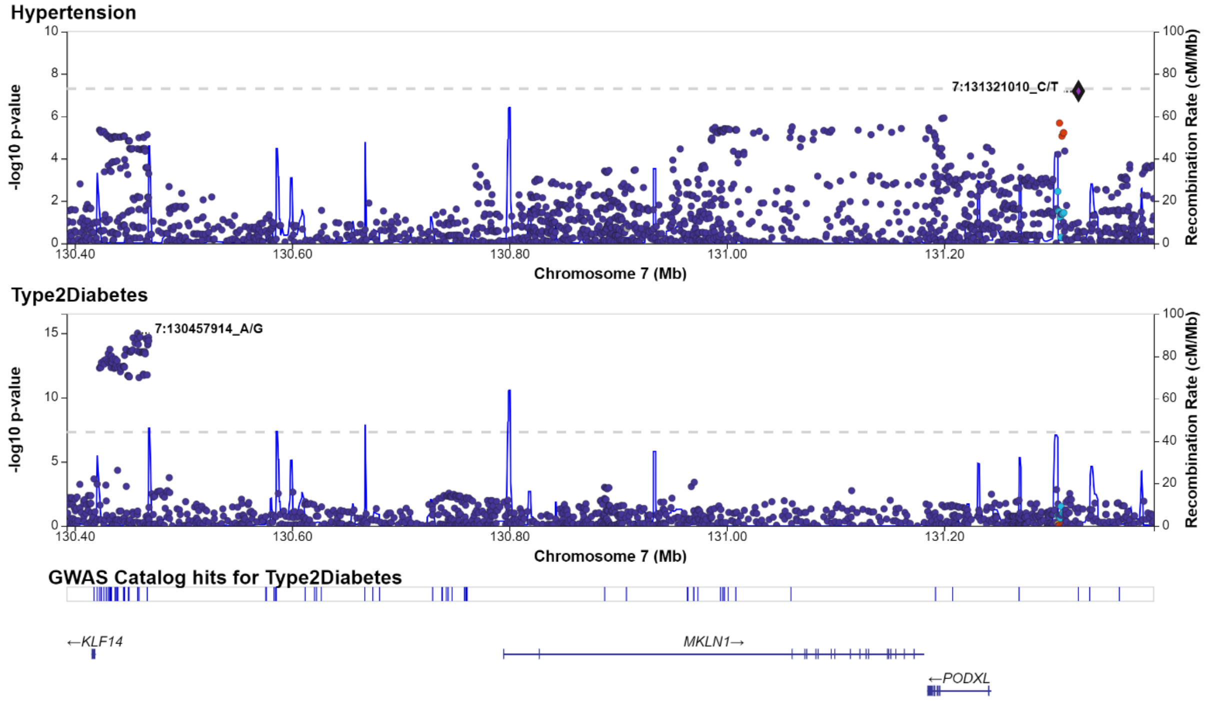

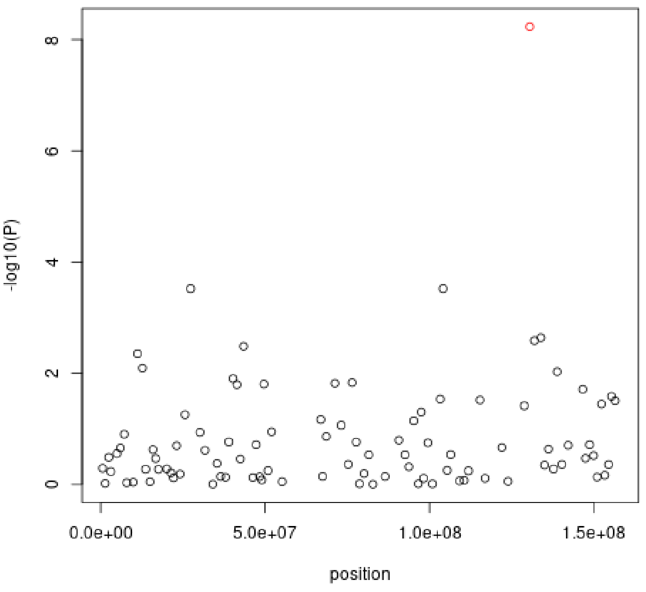

LAVA region	LAVA r_g	LAVA P	Coloc H3 PP	Coloc H4 PP	SNP Trait 1	position	P	SNP trait 2	position	P
Chr7:130418705-131856481	0.570	5.85E-09	0.997	1.1E-04	rs75672964	131321010	6.87E-08	rs1562396	130457914	9.64E-16

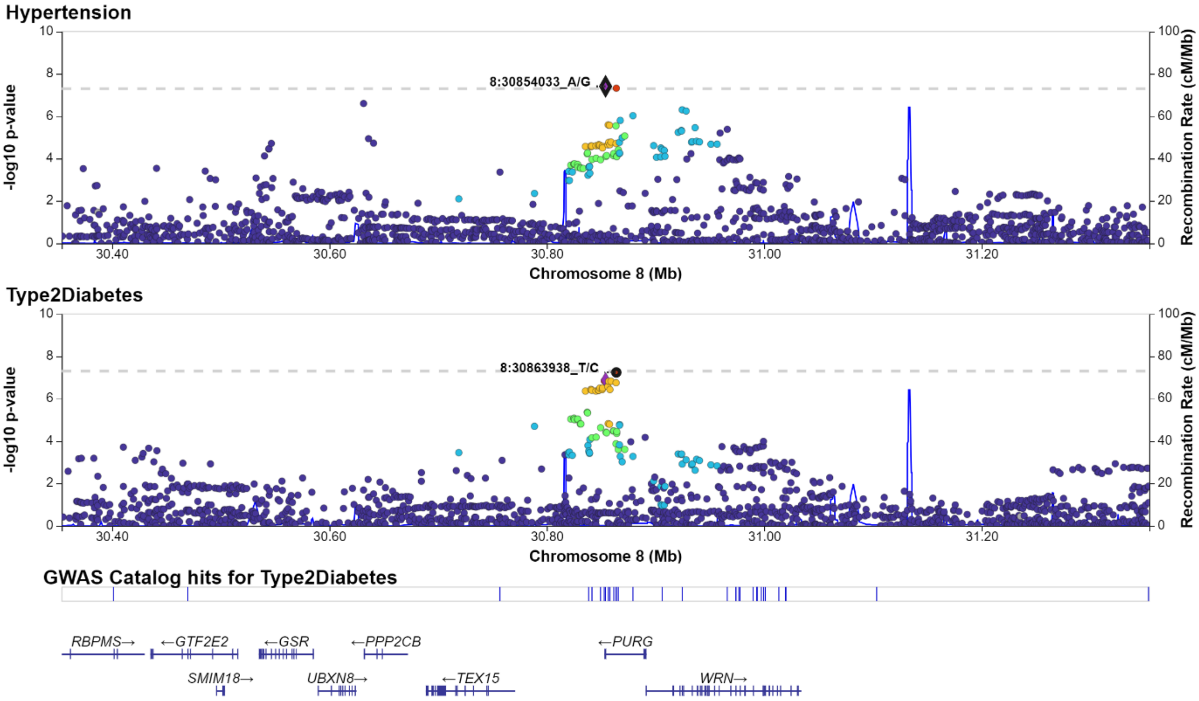

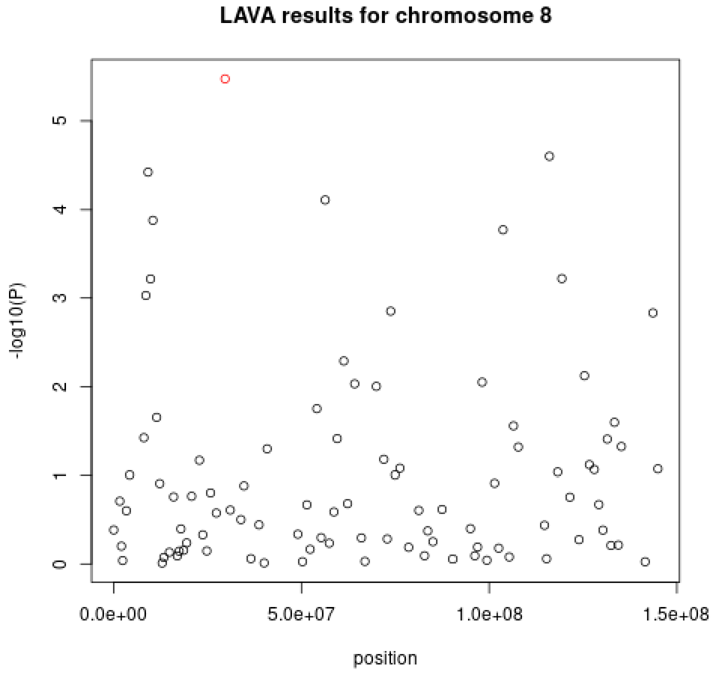

LAVA region	LAVA r_g	LAVA P	Coloc H3 PP	Coloc H4 PP	SNP Trait 1	position	P	SNP trait 2	position	P
Chr8:29744541-31134786	0.776	3.34E-06	0.013	0.986	rs2725371	30854033	3.90E-08	rs10954772	30863938	5.74E-08

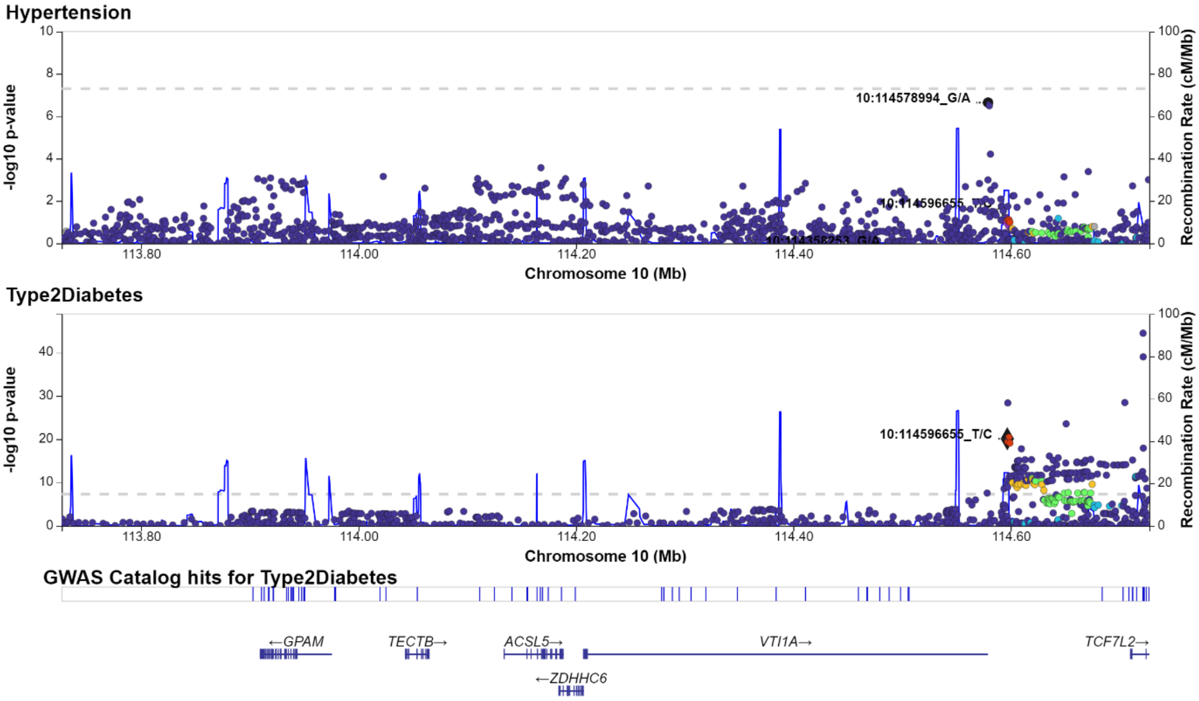
Hypertension
-log10 p-value
Recombination Rate (cM/Mb)
10:114578994_G/A
10:114596655_T/C
Chromosome 10 (Mb)
Type2Diabetes
10:114596655_T/C
GWAS Catalog hits for Type2Diabetes
←GPAM
TECTB→
ACSL5→
VTI1A→
TCF7L2→
←ZDHHC6

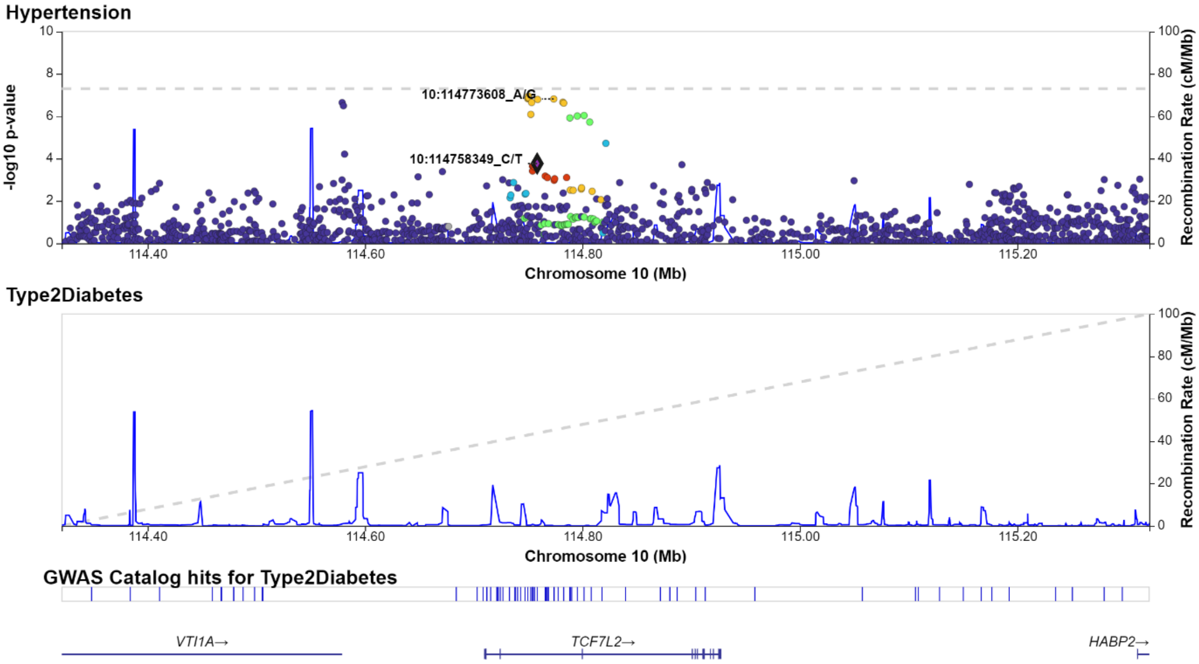
Hypertension
-log10 p-value
Recombination Rate (cM/Mb)
10:114773608_A/G
10:114758349_C/T
Chromosome 10 (Mb)
Type2Diabetes
GWAS Catalog hits for Type2Diabetes
VTI1A→
TCF7L2→
HABP2→

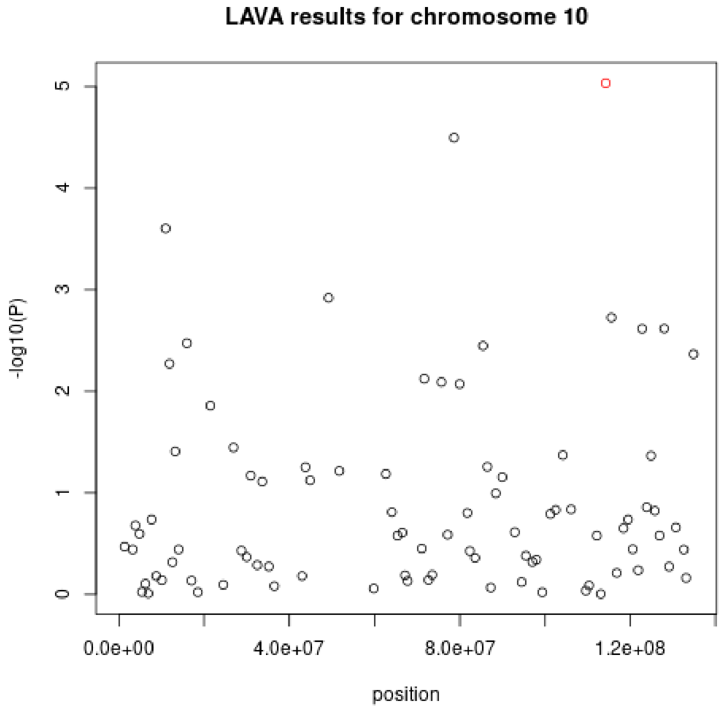

LAVA region	LAVA r_g	LAVA P	Coloc H3 PP	Coloc H4 PP	SNP Trait 1	position	P	SNP trait 2	position	P
Chr10:114255955-115588903	0.287	9.28E-06	0.994	8.96E-05	rs2479124	114578994	2.28E-07	rs72824077	114596655	7.98E-21
			0.928	6.99E-02	rs55899248	114773608	1.53E-07	rs7903146	114758349	<2.3E-319

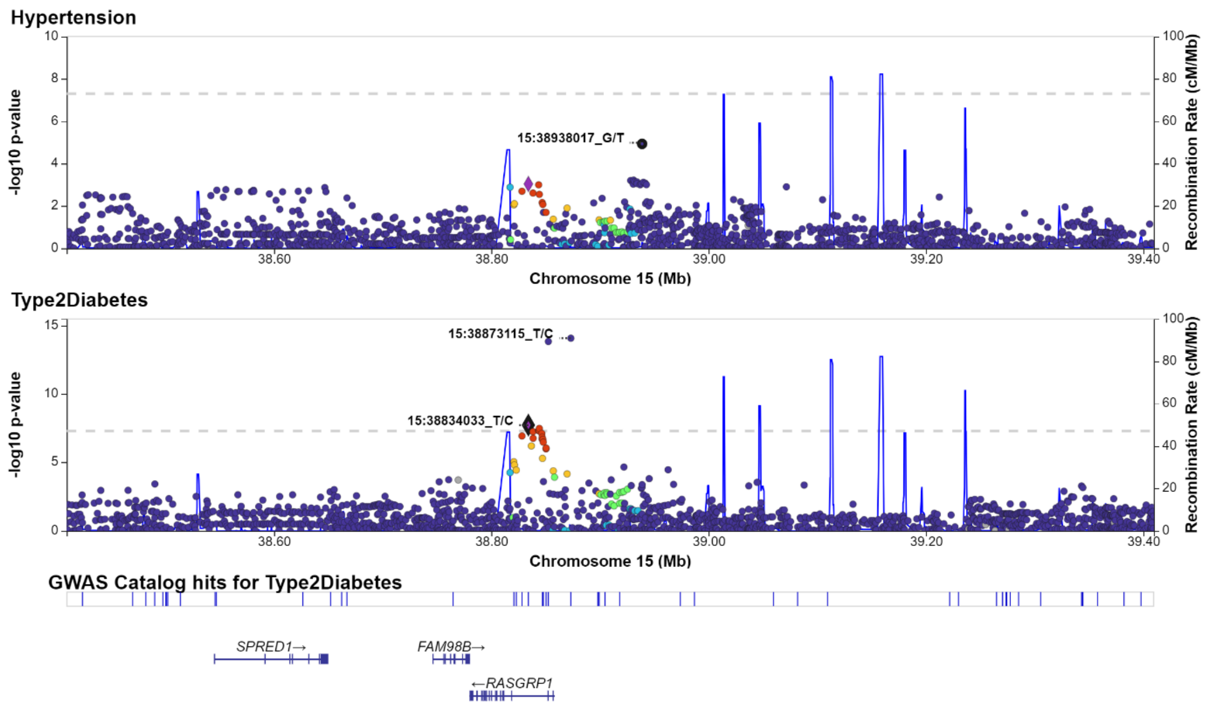

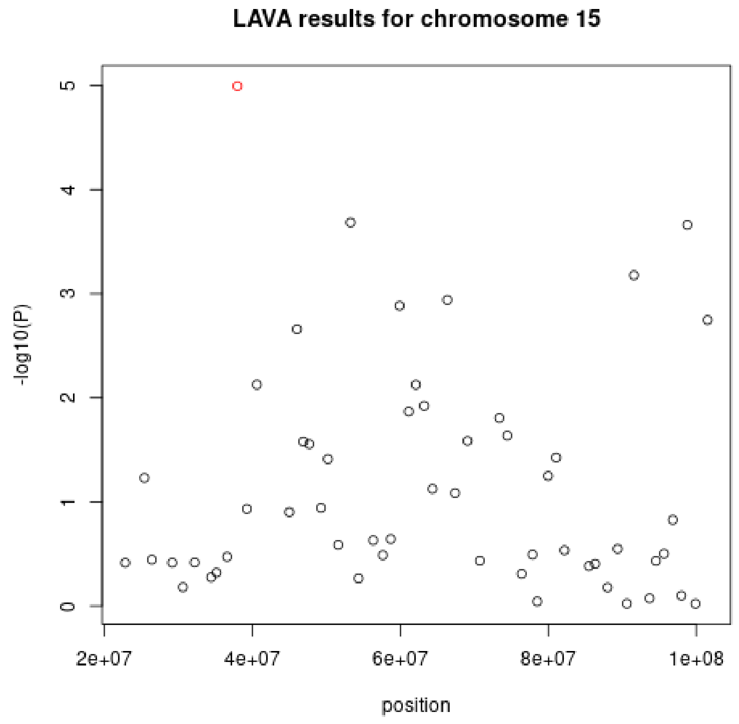

LAVA region	LAVA r_g	LAVA P	Coloc H3 PP	Coloc H4 PP	SNP Trait 1	position	P	SNP trait 2	position	P
Chr15:37962916-39238840	0.654	1.01E-05	0.430	0.015	rs10438404	38938017	1.04E-05	rs34715063	38873115	8.40E-15
			0.248	0.432	rs10438404	38938017	1.04E-05	rs8032939	38834033	1.88E-08

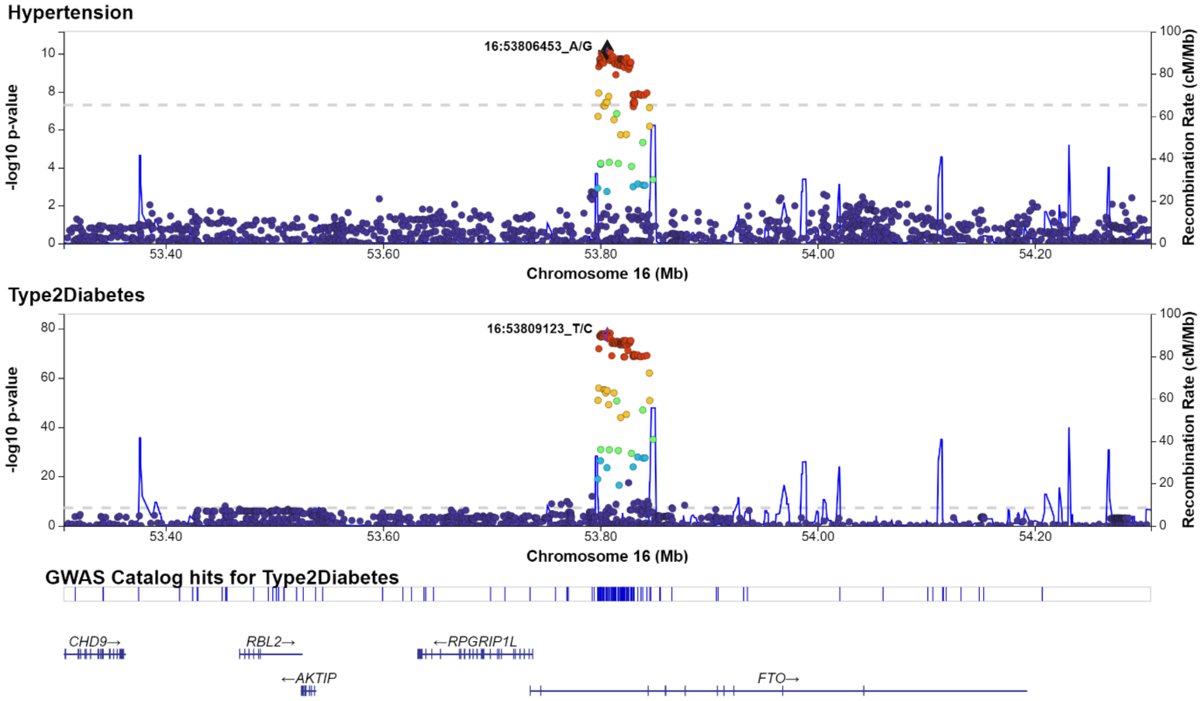

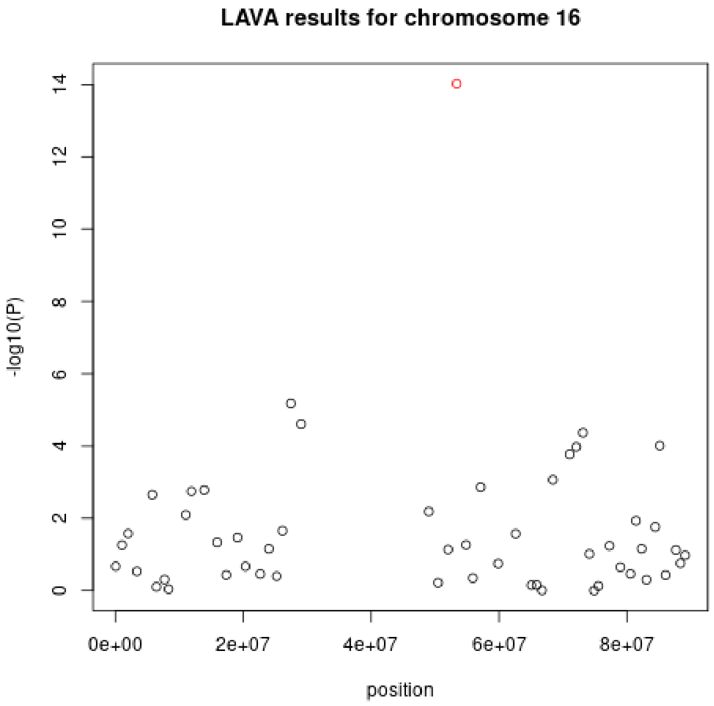

LAVA region	LAVA r_g	LAVA P	Coloc H3 PP	Coloc H4 PP	SNP Trait 1	position	P	SNP trait 2	position	P
Chr16:53393883-54866095	0.682	9.52E-15	6.00E-02	0.940	rs56094641	53806453	6.80E-11	rs55872725	53809123	8.51E-79

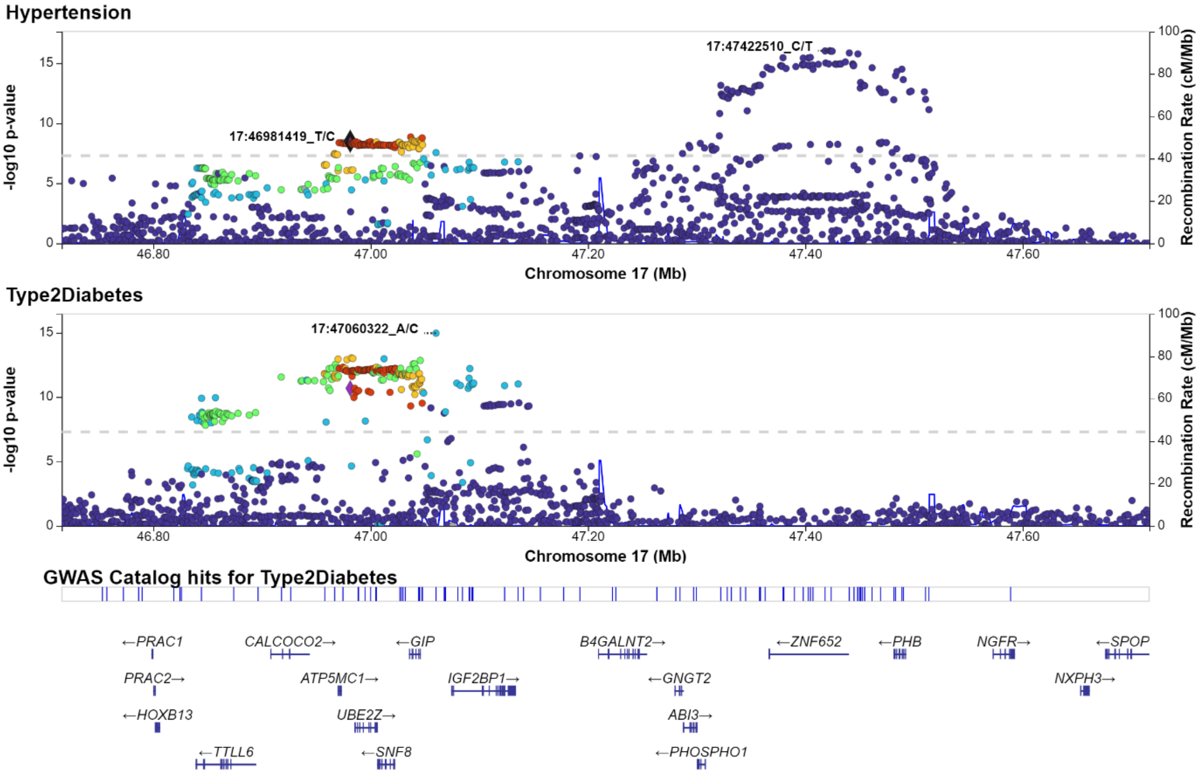

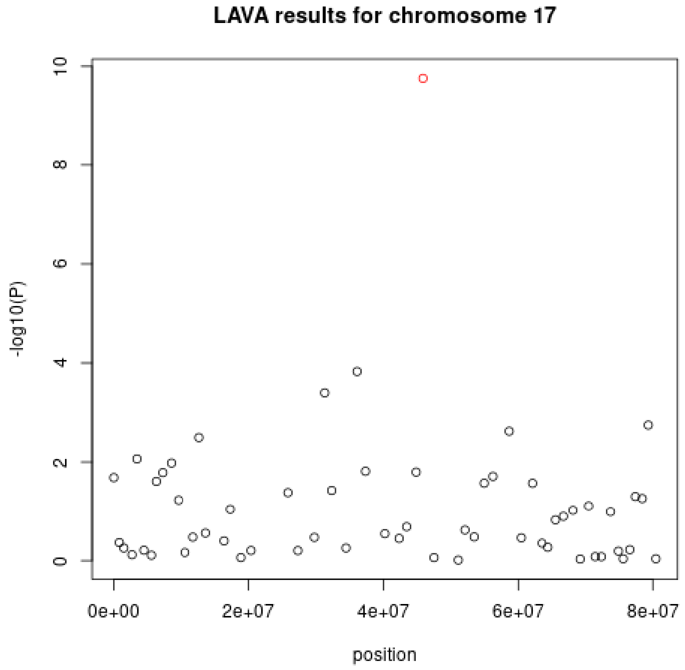

LAVA region	LAVA r_g	LAVA P	Coloc H3 PP	Coloc H4 PP	SNP Trait 1	position	P	SNP trait 2	position	P
Chr17:45883902-47516224	0.773	1.75E-10	0.960	3.99E-02	rs12940898	46981419	3.12E-09	rs35895680	47060322	1.08E-15
			1	1.68E-10	rs35073649	47422510	8.93E-17	rs35895680	47060322	1.08E-15
