# Supplementary material for: Exploring Similarities and Differences Between Methods That Exploit Patterns of Local Genetic Correlation to Identify Shared Causal Loci Through Application to Genome‐Wide Association Studies of Multiple Long Term Conditions
Source: Genet Epidemiol. 2025 Jun 19;49(5):e70012. doi: 10.1002/gepi.70012 (PMC12179580; doi:10.1002/gepi.70012)
Supplement: Supplementary file 8 — Supporting Figure S8: LocusZoom plot and coloc results for new locus identified by HDL‐L. [file GEPI-49-0-s002.pdf]

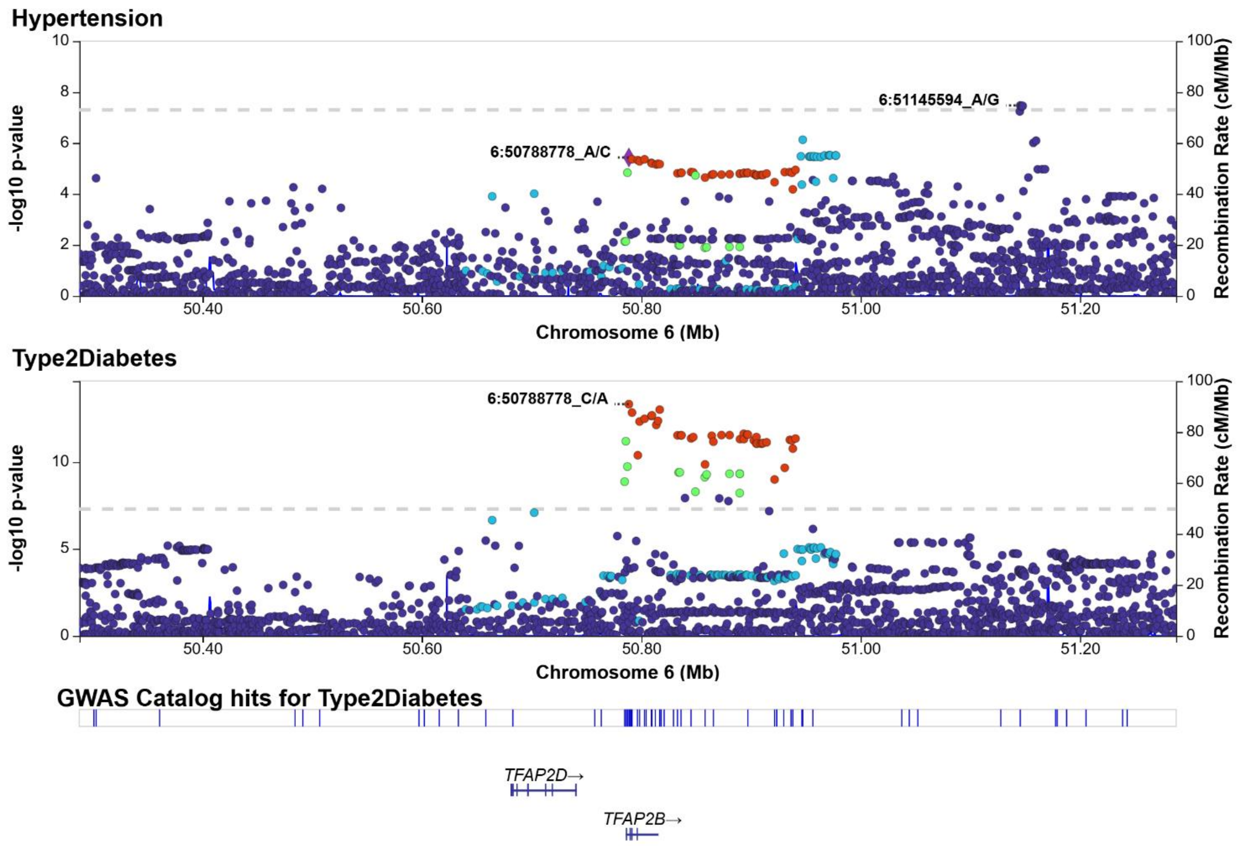

LAVA region	LAVA r_g	LAVA P	Coloc H3 PP	Coloc H4 PP	SNP Hypertension	position	P	SNP Diabetes	position	P	R^2	Gene
Chr6: 49528165-50940767	1	1.55E-04	0.034	0.964	rs3798519	50788778	6.72E-06	rs3798519	50788778	4.60E-14	1	TFAP2B
