# Supplementary material for: Exploring Similarities and Differences Between Methods That Exploit Patterns of Local Genetic Correlation to Identify Shared Causal Loci Through Application to Genome‐Wide Association Studies of Multiple Long Term Conditions
Source: Genet Epidemiol. 2025 Jun 19;49(5):e70012. doi: 10.1002/gepi.70012 (PMC12179580; doi:10.1002/gepi.70012)
Supplement: Supplementary file 9 — Supporting Table S1: Listing of the GWAS summary statistics used and their sample sizes. [file GEPI-49-0-s001.docx]

| Phenotype | Abbreviation | Cases | Controls | SNPs | Source |
| --- | --- | --- | --- | --- | --- |
| Hyptertension | HT | 84910 | 367354 | 8081164 | <http://geneatlas.roslin.ed.ac.uk/downloads/?traits=266> |
| Atrial Fibrillation | AF | 60620 | 970216 | 11427610 | <https://csg.sph.umich.edu/willer/public/afib2018/> |
| Chronic Kidney Disease | CKD | 41395 | 439303 | 8930073 | <https://ckdgen.imbi.uni-freiburg.de/datasets/Wuttke_2019> |
| Type 2 Diabetes | T2D | 80154 | 853816 | 8735477 | <https://diagram-consortium.org/downloads.html> |

Supplementary Table S1: Listing of the GWAS summary statistics used and their sample sizes
