# Supplementary material for: Exploring Similarities and Differences Between Methods That Exploit Patterns of Local Genetic Correlation to Identify Shared Causal Loci Through Application to Genome‐Wide Association Studies of Multiple Long Term Conditions
Source: Genet Epidemiol. 2025 Jun 19;49(5):e70012. doi: 10.1002/gepi.70012 (PMC12179580; doi:10.1002/gepi.70012)
Supplement: Supplementary file 10 — Supporting Table S2: Exploration of results from LAVA analysis of hypertension and type 2 diabetes in selected regions using different partition definitions. [file GEPI-49-0-s011.docx]

| chr | start | stop | n.snps | window_size | rho | rho.lower | rho.upper | P | partition |
| --- | --- | --- | --- | --- | --- | --- | --- | --- | --- |
| 1 | 38474037 | 40200950 | 3407 | 1726913 | 0.736 | 0.509 | 1 | 1.21E-07 | LAVA |
| 1 | 38731847 | 40200567 | 2921 | 1468720 | 0.710 | 0.4769 | 0.966 | 1.49E-07 | rho-HESS |
| 1 | 39537291 | 40933221 | 2664 | 1395930 | 0.783 | 0.518 | 1 | 9.54E-07 | SUPERGNOVA |
| 3 | 11997659 | 12859209 | 1931 | 861550 | 1 | 0.718 | 1 | 4.18E-09 | LAVA |
| 3 | 11019665 | 13070799 | 4801 | 2051134 | 0.735 | 0.555 | 0.931 | 1.70E-11 | rho-HESS |
| 3 | 11221721 | 12858028 | 3804 | 1636307 | 0.838 | 0.642 | 1 | 1.24E-11 | SUPERGNOVA |
| 5 | 55221399 | 55968966 | 2019 | 747567 | 0.562 | 0.379 | 0.753 | 1.21E-07 | LAVA |
| 5 | 55417349 | 56621102 | 3110 | 1203753 | 0.722 | 0.571 | 0.882 | 3.24E-14 | rho-HESS |
| 5 | 55413961 | 55932471 | 1630 | 518510 | 0.650 | 0.484 | 0.817 | 3.16E-10 | SUPERGNOVA |
| 7 | 130418705 | 131856481 | 3222 | 1437776 | 0.571 | 0.401 | 0.745 | 5.85E-09 | LAVA |
| 7 | 130422414 | 132805848 | 5063 | 2383434 | 0.705 | 0.524 | 0.895 | 3.47E-11 | rho-HESS |
| 7 | 130797776 | 131856855 | 2477 | 1059079 | 0.469 | 0.224 | 0.750 | 4.02E-04 | SUPERGNOVA |
| 10 | 114255955 | 115588903 | 2785 | 1332948 | 0.288 | 0.164 | 0.412 | 9.28E-06 | LAVA |
| 10 | 112561493 | 115328432 | 5794 | 2766939 | 0.510 | 0.378 | 0.651 | 1.28E-12 | rho-HESS |
| 10 | 113845990 | 115698315 | 3663 | 1852325 | 0.446 | 0.348 | 0.546 | 1.44E-16 | SUPERGNOVA |
| 16 | 53393883 | 54866095 | 3545 | 1472212 | 0.683 | 0.540 | 0.840 | 9.52E-15 | LAVA |
| 16 | 53382572 | 55903774 | 6396 | 2521202 | 0.743 | 0.589 | 0.927 | 1.69E-15 | rho-HESS |
| 16 | 51703888 | 53845487 | 4573 | 2141599 | 0.603 | 0.443 | 0.784 | 7.37E-11 | SUPERGNOVA |
| 17 | 45883902 | 47516224 | 3457 | 1632322 | 0.773 | 0.582 | 0.993 | 1.75E-10 | LAVA |
| 17 | 45876022 | 47517400 | 3471 | 1641378 | 0.775 | 0.582 | 1 | 2.23E-10 | rho-HESS |
| 17 | 46828412 | 48027295 | 2203 | 1198883 | 0.554 | 0.356 | 0.770 | 1.95E-06 | SUPERGNOVA |

Supplementary Table S2: Exploration of results from LAVA analysis of hypertension and type 2 diabetes in selected regions using different partition definitions
