# Supplementary material for: Exploring Similarities and Differences Between Methods That Exploit Patterns of Local Genetic Correlation to Identify Shared Causal Loci Through Application to Genome‐Wide Association Studies of Multiple Long Term Conditions
Source: Genet Epidemiol. 2025 Jun 19;49(5):e70012. doi: 10.1002/gepi.70012 (PMC12179580; doi:10.1002/gepi.70012)
Supplement: Supplementary file 11 — Supporting Table S3: Results of local correlation analysis of the simulated traits in the regions where each trait pair shares two identical causal SNPs. rg indicates the estimated local genetic correlation. [file GEPI-49-0-s006.docx]

| Trait pair | SNP | LAVA results | | ρ-HESS results | | SUPERGNOVA results | | LOGODetect results | |
| --- | --- | --- | --- | --- | --- | --- | --- | --- | --- |
|  |  | r_g_ | P | r_g_ | P | r_g_ | P | Q(R) | P |
| A+B | 10:15293155:C:T | 0.952 | 6.76E-78 | 0.004 | 1.09E-14 | 0.002 | 0.23 | 46.778 | 2E-04 |
| A+C | 2:135069472:G:A | -0.983 | 1.29E-64 | -0.010 | 2.13E-48 | -0.008 | 0.01 | -23.392 | 2E-04 |
| B+C | 12:119845498:T:A | 0.945 | 3.46E-19 | 0.003 | 3.50E-12 | 0.003 | 5.98E-04 | 25.617 | 2E-04 |
| A+B | 1:76076390:G:A | -0.919 | 5.50E-13 | -0.004 | 5.81E-10 | -0.003 | 1.92E-05 | NA | NA |
| A+C | 1:76076390:G:A | 0.994 | 1.25E-85 | 0.012 | 3.22E-59 | 0.007 | 1.56E-04 | 99.289 | 2E-04 |
| B+C | 1:76076390:G:A | -0.882 | 2.23E-12 | -0.004 | 9.28E-11 | -0.003 | 3.24E-05 | NA | NA |

Supplementary Table S3: Results of local correlation analysis of the simulated traits in the regions where each trait pair shares two identical causal SNPs. r_g_ indicates the estimated local genetic correlation.
