# Supplementary material for: Exploring Similarities and Differences Between Methods That Exploit Patterns of Local Genetic Correlation to Identify Shared Causal Loci Through Application to Genome‐Wide Association Studies of Multiple Long Term Conditions
Source: Genet Epidemiol. 2025 Jun 19;49(5):e70012. doi: 10.1002/gepi.70012 (PMC12179580; doi:10.1002/gepi.70012)
Supplement: Supplementary file 12 — Supporting Table S4: Results of local correlation analysis of the simulated traits in the regions where there is a causal SNP in the same region for two traits, but those causal SNPs are in no or very low LD with each other. rg indicates the estimated local genetic correlation. R 2 indicates the correlation between the relevant causal SNPs, as calculated by PLINK in the 1000 Genomes European reference population. [file GEPI-49-0-s009.docx]

| Trait | Chr | Causal SNP | BP | R^2^ | LAVA results | | ρ-HESS results | | SUPERGNOVA results | | LOGODetect | |
| --- | --- | --- | --- | --- | --- | --- | --- | --- | --- | --- | --- | --- |
|  |  |  |  |  | r_g_ | P | r_g_ | P | r_g_ | P | Q(R) | P |
| A  B | 22  22 | rs738409  rs5764296 | 44324727  44181312 | 0.003 | -0.220 | 0.144 | 3.55E-04 | 0.513 | 3.97E-04 | 0.009 | -3.062 | 0.001 |
| A  C | 19  19 | rs2304128  rs10412710 | 19746151  19993507 | 0.032 | 0.160 | 0.286 | 6.77E-04 | 0.225 | 5.42E-04 | 0.002 | NA | NA |
| B  C | 2  2 | rs1659676  rs1260326 | 27399294  27730940 | 0.175 | 0.443 | 2.51E-06 | 0.003 | 2.66E-05 | 0.005 | 9.14E-11 | 24.052 | 2E-04 |
| A  B | 4  4 | rs3775226  rs13118664 | 87989465  88239609 | 0.145 | -0.495 | 7.04E-06 | -0.002 | 8.57E-04 | -0.002 | 0.011 | -11.937 | 2E-04 |
| A  C | 4  4 | rs3775226  rs4693818 | 87989465  88421277 | 0.002 | 0.052 | 0.560 | -4.18E-05 | 0.947 | 0.001 | 0.016 | 15.646 | 2E-04 |
| B  C | 4  4 | rs13118664  rs4693818 | 88239609  88421277 | 0.029 | 0.065 | 0.597 | -5.5E-04 | 0.315 | -5.2E-04 | 0.196 | -4.412 | 2E-04 |

Supplementary Table S4: Results of local correlation analysis of the simulated traits in the regions where there is a causal SNP in the same region for two traits, but those causal SNPs are in no or very low LD with each other. r_g_ indicates the estimated local genetic correlation. R^2^ indicates the correlation between the relevant causal SNPs, as calculated by PLINK in the 1000 Genomes European reference population.
