# Supplementary material for: Exploring Similarities and Differences Between Methods That Exploit Patterns of Local Genetic Correlation to Identify Shared Causal Loci Through Application to Genome‐Wide Association Studies of Multiple Long Term Conditions
Source: Genet Epidemiol. 2025 Jun 19;49(5):e70012. doi: 10.1002/gepi.70012 (PMC12179580; doi:10.1002/gepi.70012)
Supplement: Supplementary file 13 — Supporting Table S5: Coloc results for regions identified via a SNP‐based screening approach. Results shown on separate lines correspond to situations where coloc identified several smaller but separate colocalising regions within the larger window. Results where the causal variant was deemed to be shared between the traits (coloc H4 posterior probability PP.H4 > 0.7) are marked in bold. [file GEPI-49-0-s004.docx]

| Trait Pair | LAVA partition | Chr | Start | End | n.snps | SNP Trait 1 | SNP trait 2 | PP.H3 | PP.H4 | LAVA P | Notes |
| --- | --- | --- | --- | --- | --- | --- | --- | --- | --- | --- | --- |
| HT-AF | 13 | **1** | **10291004** | **11296197** | **2492** | **rs17035646** | **rs284277** | **3.45E-02** | **0.965** | **8.96E-04** | **Identified by LOGODetect** |
|  | 540 | 3 | 135315218 | 136611230 | 1931 | rs1145106  rs1145106  rs1145106  rs1145106  rs1145106 | rs1279831  rs7349597  rs1278493  rs642075  rs7372313 | 0.999  0.999  0.999  0.999  0.999 | 1.36E-04  1.67E-04  2.36E-04  1.25E-04  2.06E-04 | 2.24E-02 | runsusie(AF) converged after 10000 iterations |
|  | 871 | 5 | 113872567 | 114900128 | - |  |  |  |  | 3.07E-03 | runsusie(AF) will not converge to credible sets |
|  | 884 | 5 | 127320125 | 128511507 | - |  |  |  |  | 5.43E-05 | runsusie(AF) will not converge to credible sets |
|  | 1198 | 7 | 115691315 | 117027432 | 2631 | rs56403542  rs56403542  rs56403542  rs56403542  rs56403542 | rs116928768  rs62469028  rs6954077  rs55701446  rs28557111 | 0.998  0.998  0.998  0.998  0.998 | 2.31E-05  1.97E-05  1.94E-05  1.93E-05  1.94E-05 | 0.649 | runsusie(AF) converged after 10000 iterations |
|  | 1223 | 7 | 150163837 | 151189760 | 3164 | rs3918226  rs11763131 | rs7789146  rs7789146 | 0.999  0.959 | 8.64E-08  1.80E-03 | 0.102 |  |
|  | 1251 | 8 | 11000377 | 12324292 | 3636 | rs7011756 | rs35620480 | 0.966 | 7.88E-04 | 0.639 | runsusie(HT, coverage=0.5) |
|  | 1581 | 10 | 104352912 | 105840725 | - |  |  |  |  | 0.757 | runsusie(AF) will not converge to credible sets |
|  | 2081/  2082 | **15** | **80177596** | **81518240** | **3658** | rs7174222  **rs7174222** | rs12908004  **rs1407588** | 1  **7.59E-02** | 2.34E-12  **0.924** | 1.01E-03/ **2.94E-04** | **Runs across two LAVA windows. This region identified by LOGODetect** |
|  | 2181 | 17 | 6952988 | 8280162 | 3290 | rs7223364  rs5418  rs7223364  rs5418  rs7223364  rs5418  rs7223364  rs5418  rs7223364  rs5418  rs7223364  rs5418 | rs188773436  rs188773436  rs10153276  rs10153276  rs4384652  rs4384652  rs72835264  rs72835264  rs72841462  rs72841462  rs78744936  rs78744936 | 0.995  0.999  0.994  0.999  0.995  0.999  0.995  0.999  0.995  0.999  0.992  0.999 | 1.19E-04  9.22E-07  5.18E-04  1.05E-05  2.00E-04  3.04E-06  2.82E-04  1.30E-05  4.99E-05  9.20E-07  2.91E-03  5.80E-05 | 0.288 | runsusie(AF) converged after 10000 iterations |
|  | 2208 | 17 | 44374710 | 45467306 | 1618 | rs2678713  rs17608766  rs2678713  rs17608766 | rs145153053  rs145153053  rs169201  rs169201 | 0.957  0.101  0.957  1 | 4.19E-04  8.98E-01  6.28E-04  7.66E-15 | 5.27E-04 | runsusie(AF) converged after 10000 iterations |
|  |  |  |  |  |  |  |  |  |  |  |  |
| HT-CKD | 978 | **6** | **43307092** | **44309592** | **2565** | rs7763350  **rs9472136**  rs1358980 | rs881858  **rs881858**  rs881858 | 0.999  **0.039**  0.937 | 3.01E-05  **0.960**  1.04E-03 | 1.79E-02 | **Identified by LOGODetect** |
|  | 1084 | 6 | 160148935 | 161505203 | 3838 | rs55730499  rs9295128 | rs12205178  rs12205178 | 0.999  0.999 | 2.65E-08  1.01E-05 | 0.628 |  |
|  | 1224 | **7** | **150912020** | **151912394** | **2654** | **rs73728279**  rs73728279 | **rs10224002**  rs60814812 | **0.045**  0.951 | **0.955**  5.47E-04 | **9.82E-04** | **Identified by LOGODetect** |
|  | 1643 | **11** | **30249136** | **31259370** | **1811** | **rs963837**  rs7927974 | **rs3925584**  rs3925584 | **8.87E-03**  0.997 | **0.991**  3.96E-05 | **4.60E-05** | **Identified by LOGODetect** |
|  | 1674 | 11 | 64974442 | 66030948 | - |  |  |  |  | 0.169 | No credible sets in CKD |
|  |  |  |  |  |  |  |  |  |  |  |  |
| HT-T2D | 466 | 3 | 52623465 | 54056978 | 3083 | rs3821843  rs3774468  rs73088327 | rs891368  rs891368  rs891368 | 0.999  0.997  0.834 | 2.45E-06  2.48E-05  0.121 | 2.06E-05 | Just above the LAVA cutoff |
|  | 552 | **3** | **149566947** | **150566422** | **2389** | **rs62271373** | **rs62271373** | **7.89E-05** | **0.999** | **5.05E-03** | **missed**, **rho=0.03276,SG=0.066,LOGODetect reports, so is *technically* significant, p=0.00739** |
|  | 958 | 6 | 30622459 | 31744148 | 1889 | rs9267551 | rs1077394 | 0.791 | 0.209 | 4.0E-04 | MHC complex, filtered to P <0.1 in order to run |
|  | 966 | 6 | 32162559 | 33172636 | 1914 | rs9275160  rs3132934  rs9275160  rs3132934 | rs17612852  rs17612852  rs35445101  rs35445101 | 0.994  0.989  0.989  0.989 | 2.64E-04  6.45E-04  4.92E-03  2.22E-04 | 2.69E-05 | MHC complex, filtered HT P<0.05 AND runsusie(HT, coverage =0.5), filtered DB P<0.1 |
|  | 977/978 | **6** | **42849523** | **44258330** | **3442** | rs7763350  rs1358980  rs9472136  rs7763350  rs1358980  rs9472136  rs7763350  **rs1358980**  rs9472136 | rs6937438  rs6937438  rs6937438  rs62401198  rs62401198  rs62401198  rs11967262  **rs11967262**  rs11967262 | 0.999  0.976  0.132  0.892  0.881  0.901  0.999  **0.015**  0.998 | 2.31E-10  3.46E-04  0.868  1.31E-02  1.98E-03  1.08E-03  6.77E-07  **0.985**  2.18E-05 | 3.54E-04/0.120 | **Identified by LOGODetect, straddles LAVA window** |
|  | 1054 | 6 | 126292187 | 127655444 | 1818 | rs9372863  rs62426328  rs9372863  rs62426328 | rs11759026  rs11759026  rs10456964  rs10456964 | 0.693  1  0.703  0.999 | 1.78E-02  2.19E-10  3.81E-03  8.55E-09 | 0.529 |  |
|  | 1084 | 6 | 160270553 | 161505203 | 3427 | rs55730499  rs9295128 | rs474513  rs474513 | 0.999  0.999 | 4.24E-07  1.25E-05 | 2.9E-02 |  |
|  | 1223 | 7 | 150037927 | 151189726 | 3494 | rs3918226  rs11761125  rs11763131 | rs62492368  rs62492368  rs62492368 | 0.999  0.988  0.973 | 6.70E-06  6.78E-04  1.21E-03 | 0.303 |  |
|  | 1246 | 8 | 8072330 | 8804248 | 3235 | rs4841029 | rs11780774 | 0.446 | 0.553 | 3.8E-02 | runsusie(DB, coverage = 0.5) |
|  | 1247 | 8 | 8106406 | 9324858 | 5333 | rs330072  rs330072 | rs17662402  rs2126263 | 0.779  0.898 | 7.72E-03  2.30E-02 | 9.35E-04 | runsusie(DB,coverage = 0.5) and runsusie(HT,coverage=0.5) |
|  | 1249 | 8 | 9478234 | 10496302 | 4046 | rs615632 | rs34990153 | 0.614 | 0.368 | 6.08E03 | runsusie(DB, coverage = 0.1) |
|  | 1250 | 8 | 10086946 | 11402683 | 5038 | rs6991641 | rs2001433 | 0.442 | 0.557 | 1.32E-04 | runsusie(DB, coverage = 0.5) |
|  | 1353 | 8 | 127895694 | 129211415 | 4171 | rs62513967 | rs17772814 | 0.989 | 1.64E-04 | 8.62E-02 | runsusie(HT, coverage = 0.5) |
|  | 1398 | 9 | 21619202 | 22633841 | 2553 | rs1333045  rs1333045  rs1333045  rs1333045  rs1333045  rs1333045  rs1333045 | rs7856455  rs10757283  rs79698600  rs10811660  rs12376511  rs1575972  rs1360589 | 0.997  0.997  0.976  0.997  0.995  0.997  0.869 | 2.50E-05  4.76E-05  3.01E-04  5.02E-04  6.30E-05  2.54E-05  0.129 | 1.25E-04 |  |
|  | 1479 | 9 | 135649679 | 137035570 | 4930 | rs10993958 | rs505922 | 0.996 | 1.31E-04 | 0.150 |  |
|  | 1612 | 11 | 1388757 | 2697065 | 4446 | rs11041487  rs569550  rs74050111  rs17245346  rs11041487  rs569550  rs74050111  rs17245346  rs11041487  rs569550  rs74050111  rs17245346  rs11041487  rs569550  rs74050111  rs17245346 | rs231360  rs231360  rs231360  rs231360  rs4929965  rs4929965  rs4929965  rs4929965  rs2283169  rs2283169  rs2283169  rs2283169  rs76074250  rs76074250  rs76074250  rs76074250 | 1  1  0.999  0.932  1  1  0.999  0.933  0.999  0.999  0.999  0.931  0.999  0.999  0.999  0.931 | 3.04E-10  1.89E-14  5.91E-06  1.59E-03  2.27E-10  1.69E-14  2.99E-06  6.90E-04  1.74E-05  4.08E-05  7.70E-05  1.31E-03  8.52E-06  5.84E-06  3.01E-05  2.41E-03 | 0.012 |  |
|  | 1655 | 11 | 43120315 | 44377357 | 3582 | rs55670730 | rs1061810 | 0.413 | 0.582 | 7.76E-05 | runsusie(HT, coverage = 0.1) |
|  | 1657 | 11 | 45358607 | 46724068 | 2616 | rs72910075  rs4237662 | rs10838524  rs10838524 | 0.998  0.928 | 3.59E-05  1.40E-03 | 0.079 |  |
|  | 1658 | 11 | 46906706 | 48029285 | 1750 | rs11039216  rs11039216 | rs11604324  rs2293578 | 0.999  0.832 | 2.81E-06  0.157 | 0.845 |  |
|  | 1674 | 11 | 64795772 | 65972724 | 2040 | rs10750766 | rs1783541 | 1 | 4.26E-07 | 0.014 |  |
|  | 2050 | 15 | 40908207 | 42301497 | 2888 | rs692155 | rs2297383 | 0.998 | 2.94E-05 | 7.48E-03 |  |
|  | 2090 | 15 | 90923577 | 92010377 | 2998 | rs6224 | rs12910825 | 1 | 1.66E-11 | 0.949 |  |
|  | 2162 | 16 | 81035077 | 82098308 | 4563 | rs7199293 | rs2925979 | 0.993 | 1.01E-03 | 1.18E-02 |  |
|  | 2172 | 16 | 89064420 | 90171343 | 3819 | rs6500460 | rs12920022 | 0.989 | 2.83E-04 | 0.106 | runsusie(HT, coverage = 0.5) |
|  | 2312 | 19 | 4451560 | 5470410 | 3255 | rs12609484 | rs262549 | 0.994 | 1.90E-03 | 1.19E-03 | runsusie(HT, coverage = 0.1) |
|  | 2406 | 20 | 42295379 | 43503038 | 2897 | rs6031431  rs6031431  rs6031431  rs6031431  rs6031431  rs6031431  rs6031431 | rs1800961  rs76811102  rs244080  rs36112520  rs11696357  rs4812770  rs285205 | 1  0.997  0.981  1  0.999  0.998  0.981 | 1.60E-11  3.17E-05  1.72E-04  5.75E-11  6.70E-08  2.23E-05  2.42E-04 | 0.607 |  |
|  | 2422 | 20 | 56897913 | 58239524 | 3508 | rs73306876 | rs4812034 | 0.999 | 1.69E-08 | 0.416 |  |

Supplementary Table S5: coloc results for regions identified via a SNP-based screening approach. Results shown on separate lines correspond to situations where coloc identified several smaller but separate colocalising regions within the larger window. Results where the causal variant was deemed to be shared between the traits (coloc H4 posterior probability PP.H4 > 0.7) are marked in bold.
