# Supplementary material for: Exploring Similarities and Differences Between Methods That Exploit Patterns of Local Genetic Correlation to Identify Shared Causal Loci Through Application to Genome‐Wide Association Studies of Multiple Long Term Conditions
Source: Genet Epidemiol. 2025 Jun 19;49(5):e70012. doi: 10.1002/gepi.70012 (PMC12179580; doi:10.1002/gepi.70012)
Supplement: Supplementary file 14 — ADMISSION Research Collaborative (29th July 2024). [file GEPI-49-0-s003.docx]

**ADMISSION Research Collaborative (29^th^ July 2024)**

Victoria Bartle^1^

Rachel Cooper^2,3^

Heather J Cordell^4^

Ray Holding^1^

Tom Marshall^5^

Fiona E Matthews^6^

Paolo Missier^7^

Ewan R Pearson^8^

Chris Plummer^3,9^

Sian M Robinson^2,3^

Elizabeth Sapey^10, 11^

Thomas Scharf^4^

Avan A Sayer^2,3^

Mervyn Singer^12,13^

James MS Wason^14^

Miles D Witham^2,3^

1. Public Co-Investigator, ADMISSION Research Collaborative, Newcastle upon Tyne, UK
2. AGE Research Group, Translational and Clinical Research Institute, Faculty of Medical Sciences, Newcastle University, Newcastle upon Tyne, UK
3. NIHR Newcastle Biomedical Research Centre, Newcastle upon Tyne Hospitals NHS Foundation Trust, Cumbria, Northumberland, Tyne and Wear NHS Foundation Trust and Newcastle University, Newcastle upon Tyne, UK
4. Population Health Sciences Institute, Faculty of Medical Sciences, Newcastle University, Newcastle upon Tyne, UK
5. Institute of Applied Health Research, University of Birmingham, Birmingham, UK
6. Research and Enterprise Office, University of Hull, Hull, UK
7. School of Computer Science, University of Birmingham, Birmingham, UK
8. Division of Population Health and Genomics, Ninewells Hospital and School of Medicine, University of Dundee, Dundee, UK
9. Digital Services, Newcastle upon Tyne Hospitals NHS Foundation Trust, Newcastle upon Tyne, UK
10. PIONEER Hub, University of Birmingham, Birmingham, UK.
11. Institute of Inflammation and Ageing, University of Birmingham, Birmingham, UK.
12. University College London Hospitals NHS Foundation Trust, London, UK
13. Bloomsbury Institute for Intensive Care Medicine, University College London, London, UK
14. Biostatistics Research Group, Population Health Sciences Institute, Newcastle University, Newcastle upon Tyne, UK
